# SIX1^+^PAX3^+^ identify a progenitor for myogenic lineage commitment from hPSCs

**DOI:** 10.1242/dev.201509

**Published:** 2023-07-13

**Authors:** Olga G. Jaime, Jessica Arias, Shreya Pavani, April D. Pyle, Michael R. Hicks

**Affiliations:** ^1^ Department of Physiology and Biophysics, School of Medicine, University of California, Irvine, Irvine, CA 92697, USA; ^2^ Sue and Bill Gross Stem Cell Research Center, University of California, Irvine, Irvine, CA 92697, USA; ^3^ Microbiology, Immunology, and Molecular Genetics, University of California, Los Angeles, CA 90095, USA

**Keywords:** Directed differentiation, Myogenesis, Pluripotent stem cells, SIX1, Skeletal muscle, Human

## Abstract

The earliest skeletal muscle progenitor cells (SMPCs) derived from human pluripotent stem cells (hPSCs) are often identified by factors expressed by a diverse number of progenitors. An early transcriptional checkpoint that defines myogenic commitment could improve hPSC differentiation to skeletal muscle. Analysis of several myogenic factors in human embryos and early hPSC differentiations found SIX1^+^PAX3^+^ co-expression was most indictive of myogenesis. Using dCas9-KRAB hPSCs, we demonstrate that early inhibition of *SIX1* alone significantly decreased *PAX3* expression, reduced PAX7^+^ SMPCs, and myotubes later in differentiation. Emergence of SIX1^+^PAX3^+^ precursors can be improved by manipulating seeding density, monitoring metabolic secretion and altering the concentration of CHIR99021. These modifications resulted in the co-emergence of hPSC-derived sclerotome, cardiac and neural crest that we hypothesized enhanced hPSC myogenic differentiation. Inhibition of non-myogenic lineages modulated *PAX3* independent of *SIX1*. To better understand SIX1 expression, we compared directed differentiations to fetal progenitors and adult satellite cells by RNA-seq. Although SIX1 continued to be expressed across human development, SIX1 co-factor expression was dependent on developmental timing. We provide a resource to enable efficient derivation of skeletal muscle from hPSCs.

## Teaser

**Summary:** This study defines specific steps to generate more robust and predictable myogenic cells from human pluripotent stem cells and explores the regulation of SIX1 in early myogenic commitment.

## INTRODUCTION

Directed differentiation of human pluripotent stem cells (hPSCs) is a process that follows developmental cues to form cell lineages which can be used for personalized therapies or to improve our understanding of human biology. The use of stepwise protocols that mimic sequential developmental signals have already impacted translational stem cell biology across many fields. For example, directed differentiation of hPSCs to pancreatic cells first described the generation of fetal-like β-progenitors (Pagliuca et al., 2014) that were further matured to generate insulin producing islet cells now used in treatments for Type 1 diabetes (Sharon et al., 2019). The directed differentiation to skeletal muscle has not yet reached clinical trial stages, although many labs have demonstrated the generation of fetal-like skeletal muscle progenitor cells (SMPCs) and myotubes *in vitro* (Borchin et al., 2013; Chal et al., 2016; Choi et al., 2016; Hicks et al., 2018; Shelton et al., 2016; Uchimura et al., 2017; Xi et al., 2017). Several challenges remain in the derivation of hPSCs to muscle which diminish translational ability. Not all labs have been able to replicate these directed differentiation protocols (Wu et al., 2018) and we do not fully understand how hPSCs transition from the somite to form the dermomyotome and myotome regions. The vast majority of published hPSC differentiation protocols each produce unique and impure cultures containing non-myogenic cell lineages (Xi et al., 2020), which may support or inhibit myogenic commitment and/or affect the long-term maintenance of PAX7^+^ SMPCs (Nalbandian et al., 2021). A better understanding of these challenges is an important step in generating clinical-grade skeletal muscle cells from hPSCs.

Genetic variability further complicates the modeling of development and disease from hPSCs (Boulting et al., 2011; Doss and Sachinidis, 2019). Using a standard protocol to differentiate motor neurons, seminal research has demonstrated line-to-line heterogeneity, even though poorly differentiated lines expressed pluripotency markers and retained ability to form teratomas (Boulting et al., 2011). The differentiation potential amongst patient-specific hPSCs may be non-constant owing to variations in cell proliferation, metabolism and response to small molecules (Osafune et al., 2008). Because of genotypic heterogeneity, the need to make tailored modifications to directed differentiations is imperative for effective lineage specification.

A better understanding of early myogenic commitment in humans is essential to understand how to efficiently direct hPSCs onto the skeletal muscle lineage. In model organisms, the genetic programs that precede the formation of mature myofibers have been well studied; however, skeletal myogenesis in humans is complex and limited studies exist on prenatal muscle development that can inform on hPSC differentiation. The origin of skeletal muscle traces back to the paraxial mesoderm, which gives rise of the presomitic mesoderm and the somite. Groups have developed hPSC derivation protocols that use a combination of small molecules to recapitulate presomitic mesoderm and somite stages (Chal et al., 2015; Xi et al., 2017). However, after compartmentalization of the somite into the ventral sclerotome and dorsal dermomyotome, it is the dermomyotome that goes on to form skeletal muscle (Chal and Pourquie, 2017), and less is known about the derivation of these structures from hPSCs.

Key regulators of myogenic specification and organ development *in vivo* is reliant on the Pax family of transcription factors Pax3 and Pax7. However, before progenitors homing to the dermomyotome, Pax genes are not restricted to the myogenic lineage, but are also detected in the dorsal region of the neural tube (Fougerousse et al., 2002) and in lateral plate where neural crest progenitors arise (Boudjadi et al., 2018; Buckingham and Relaix, 2015). Induction of skeletal myogenesis also involves the coordination of transcription factors Meox1, Six1 and its cofactors Eya1/2 (Ridgeway and Skerjanc, 2001). In the mouse embryo, Six1^−/−^ mutants lack a diaphragm and are devoid of specific limb muscles, while both Eya1^−/−^Eya2^−/−^ and Six1^−/−^Six4^−/−^ mutants show severe downregulation of Pax3 in the hypaxial dermomyotome from which cMet^+^ (also known as MET) migrating progenitors originate, leading to migratory defects and limbs lacking muscle (Grifone et al., 2007). These factors have been shown to be expressed during human skeletal myogenesis (Fougerousse et al., 2002), but the expression and timing of these genes during hPSC differentiation to muscle are unclear.

During development, the co-emergence of non-myogenic cell lineages is known to produce factors that regulate skeletal muscle specification *in vivo*. For example, the somite also matures to form the sclerotome compartment giving rise to SOX9^+^ cartilage progenitors. Sox9 mutations in mice cause limb defects with abnormal skeletal muscle formation, whereas SOX9 mutations in humans cause severe skeletal dysmorphology, campomelic dysplasia (Akiyama et al., 2002). In addition, the somite and its myogenic derivatives develop adjacent to neural crest cells. Studies have demonstrated that *Sox10* neural crest cells provide survival cues for developing SMPCs and potentially modulate early myogenic cell fate decisions (Rios et al., 2011; Van Ho et al., 2011). These lineages also arise during hPSC differentiation, but their interactions with SMPCs *in vitro* are unclear, and whether their ability to consistently form across multiple hPSC lines has not been investigated.

Besides the myogenic transcriptional programs, a plethora of signaling mechanisms in surrounding tissues such as the ectoderm and neural tube are involved in SMPC specification; amongst these, Wnt signaling is of high importance (Tajbakhsh and Buckingham, 2000). Multiple Wnt ligands and receptors participate in all steps of embryonic muscle development including somite segmentation, dermomyotome specification and subsequent myogenic differentiation (Girardi and Le Grand, 2018). These signals are elegantly organized to regulate emergence of myogenic regulatory factors, thus precise temporal and dose-dependent exposure of Wnt signals can drive differential mesoderm cell fate decisions (Loh et al., 2016). As such, small changes in Wnt signaling during *in vitro* differentiation of skeletal muscle may induce specification of other mesodermal lineages and increase the expression of ISL1^+^ progenitors (Zhao et al., 2019). The combination of myogenic factors, multiple cell lineages and signaling mechanisms may lead to variability in the efficiency to direct hPSCs to skeletal muscle.

Here, we define a stepwise approach to modify differentiation for several hPSC lines to efficiently derive SMPCs. Although SMPCs and myotubes require 4-7 weeks to differentiate, we characterize a key developmental checkpoint at an early stage of myogenic commitment after 9 days of differentiation which can predict successful hPSC differentiation to SMPCs. We demonstrate how to optimize the directed differentiation from multiple patient-specific human induced pluripotent stem cell (hiPSC) and human embryonic stem cell (hESC) lines at varying ages and backgrounds and validate the emergence of myogenic-specific markers SIX1 and PAX3 early during the differentiation protocol. ERBB3^+^NGFR^+^ can be used to enrich and identify PAX7^+^ myogenic populations in fetal cells and hPSCs (Hicks et al., 2018); therefore, we used these markers to measure positive skeletal muscle differentiation and demonstrate the requirement of *SIX1* in regulating early skeletal muscle commitment. We further explore the role of non-myogenic lineages during *in vitro* myogenesis using CRISPR/Cas9 inhibition of neural crest lineages and non-muscle mesoderm lineages and demonstrate that knockdown of these lineages can regulate PAX3 independent of SIX1 expression. The specification through temporal stages to form muscle is poorly understood and thus we performed RNA-seq on muscle stem and progenitor cells across human development to identify signals that regulate *SIX1* and myogenesis over time. This work will improve our understanding of how multiple cell types and conditions create the optimal *in vitro* microenvironment during myogenesis. These findings demonstrate that we can recapitulate prenatal myogenesis *in vitro* and can robustly differentiate hPSCs into pre-myogenic progenitors.

## RESULTS

### SIX1^+^PAX3^+^ represents an early myogenic progenitor *in vivo* and *in vitro*

**Fig. 1.**
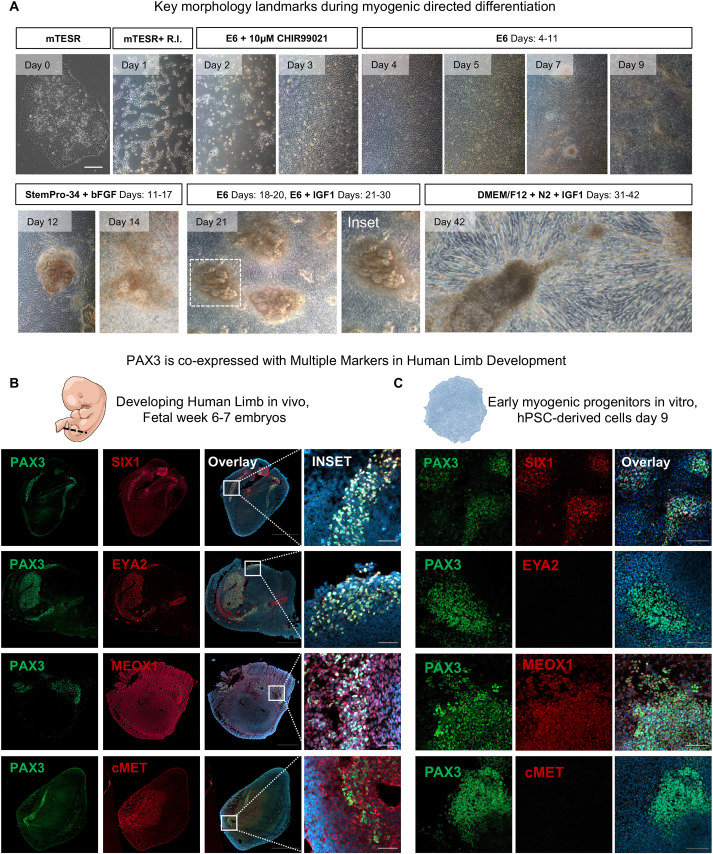
**SIX1 and PAX3 identify an early myogenic progenitor *in vivo* and *in vitro*.** (A) Overview of directed differentiation protocol highlighting key developmental stages. Morphology of cell culture shows example of pluripotent colony (H9). We chose day 9 as our checkpoint for myogenic differentiation. Please refer to supplementary Materials and Methods for detailed protocol. (B,C) Cross-section of human fetal week 6-7 limb buds (B) and day 9 cultures (C) stained with Hoechst 33342 (blue), PAX3 (green) and SIX1, EYA2, MEOX1 or cMET (red) antibodies. The limb bud length (anterior-posterior) is indicated by the dashed line in embryo illustration. Scale bars: 100 μm.

Using a previously published approach, we differentiated skeletal muscle progenitor cells (SMPCs) from hPSCs (Hicks et al., 2018). One week after mesoderm induction, most cultures gave rise to distinct 3D structures that could be identified by brightfield imaging. We also found that myotubes predominately formed juxtaposed to these structures (Fig. 1A). Thus, we reasoned that these 3D structures may regulate the formation of the earliest myogenic progenitors and selected this key morphological time point for further investigation. To evaluate myogenic specification, we compared hPSC cultures with human embryonic limb tissues at week 6-7, a time at which PAX3^+^ myogenic progenitors have delaminated from the myotome to form limb muscles. We analyzed co-expression of PAX3 with several other early myogenic markers including SIX1, its cofactor EYA2, MEOX1 and cMET. We found that PAX3 was regionalized to the periphery of tissues, suggesting that the cells were pre-limb bud muscle progenitors. Transcription factors SIX1 and EYA2 colocalized with PAX3; however, we found that both MEOX1 and cMET not only colocalized with PAX3 but also were expressed by other embryonic cell types throughout the limb (Fig. 1B). Similar to human embryonic tissue, we found that SIX1 best colocalized with PAX3 expression in hPSC-derived skeletal muscle at day 9 (Fig. 1C). These cells radially expressed SIX1^+^PAX3^+^ but did not express EYA2 nor the migratory marker cMET, suggesting that these cells did not yet interact with EYA2 or were not identical to those in the limb bud progenitors. MEOX1 was expressed on nearly all hPSC progenitors at day 9, suggesting nonspecific skeletal muscle identity. We used a second directed differentiation approach (Chal et al., 2016) and similarly demonstrated the derivation of a SIX1^+^PAX3^+^ myogenic population; We also found that both *EYA2* and *cMET* expression was absent and that the cultures highly expressed *MEOX1* this time point (Fig. S1). Thus, SIX1^+^PAX3^+^ may best represent an early emerging myogenic population *in vitro*.

### Identification of an early myogenic lineage commitment panel as a checkpoint for myogenic differentiation quality across hPSC lines

**Fig. 2.**
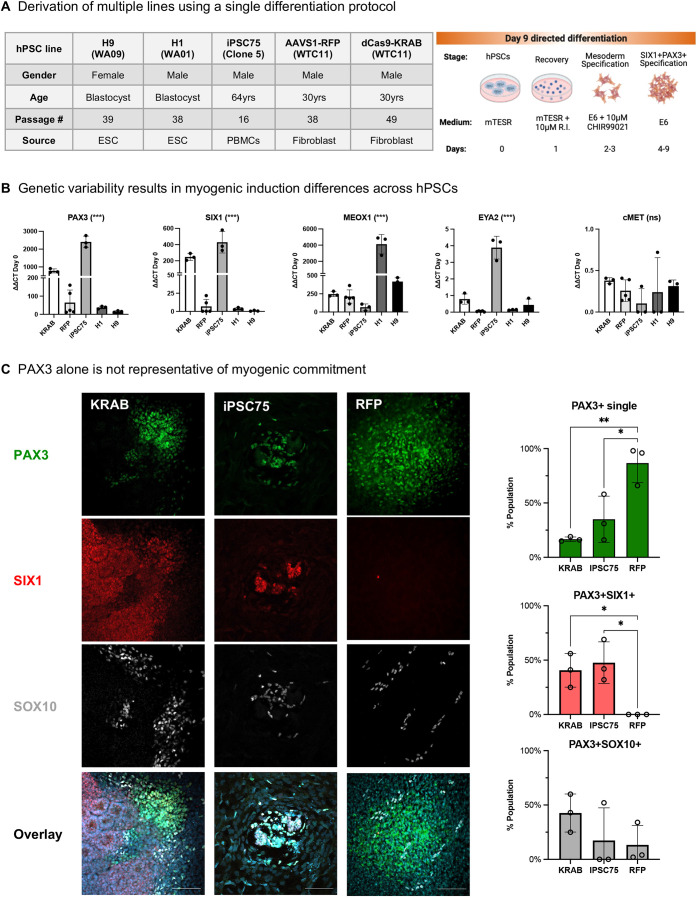
**Myogenic gene expression is variable across hPSC lines.** (A) Representative table and schematic of directed differentiation of hPSCs to skeletal muscle progenitors through day 9. (B) Quantitative RT-qPCR analysis of the various hPSC lines differentiated to SMPCs through day 9. Day 9 data normalized to *GAPDH* and relative to expression of hPSCs at day 0 (*n*=3-5 biological replicates). ***P<0.001 (one way ANOVA). (C) Day 9 cultures stained with Hoechst 33342 (blue), PAX3 (green), SIX1 (red) and SOX10 (white) antibodies. Quantification of SIX1^+^PAX3^+^ and PAX3^+^SOX10^+^ cells expressed as the total number of nuclei/mm^2^ (*n*=3 regions of SIX1^+^PAX3^+^ cells). *P<0.05, **P<0.01 (one way ANOVA). Data are mean±s.d. ns, not significant. Cells were counted on ImageJ software. Scale bars:100 μm.

The efficiency to derive SMPCs often varies by hPSC inherent differentiation capacity, which may limit the use of some hPSC lines for personalized medicine. To evaluate the consistency of early myogenic commitment, we differentiated five hPSC lines of male and female origin, including the comparison of two transgenic lines from the same background (WTC-11), to measure how clonality affected expression of key pre-myogenic genes (Fig. 2A). Although all lines showed higher levels of *PAX3* expression from hPSCs at day 0, we found up to 50-fold variation in *PAX3* across lines at this time point. Across all hPSC lines, *SIX1* followed a similar expression pattern to *PAX3*, although its expression remained lower than *PAX3*, suggesting either that *SIX1* emerges later or that *PAX3* is also expressed by non-myogenic cell types. Compared with the hiPSCs lines, both hESC lines showed signs of a poor or slower differentiation based on reduced *PAX3* and *SIX1* expression. *EYA2* and *cMET* were expressed at a low level or not at all in any hPSC-line at this timepoint, which was unlike the human embryonic tissues. We also noted an inverse correlation between the expression pattern of *PAX3* and *SIX1* with that of *MEOX1* expression*.* Although MEOX1 increased 100- to 200-fold in hiPSCs, its expression increased up to 4000-fold in hESCs, suggesting that *MEOX1* cells either successfully expand in the absence of *PAX3* and *SIX1* or that these two markers precede *MEOX1* (Fig. 2B).

To determine the myogenic identity of PAX3 expressing cells, we co-stained and quantified whether PAX3 showed co-expression with the myogenic marker SIX1 or the neural crest marker SOX10. We found that PAX3 cells mostly clustered together; however, cluster size and the number of cells within a cluster varied between the dCas9-KRAB line (550±110), the AAVS1-RFP line (300±160) and iPSC75 (120±30) per field of view (0.14 mm^2^). PAX3^+^ clusters were not indicative of SIX1 nor SOX10 co-expression, which varied between 0% and 50% with SIX1 or 15% and 50% with SOX10 co-staining from the total number of PAX3^+^ cells (Fig. 2C). Further, we found that the AAVS1-RFP line did not give rise to a SIX1^+^PAX3^+^ cell type at this time point, suggesting that the conditions we used to differentiate it were not optimal.

To determine whether this line-to-line variation was due to changes in pluripotency status, we measured SOX2 and OCT4 expression and found that all hPSC colonies were >97% double positive for these pluripotency markers (Fig. S2A). We found no correlation between ability to form muscle and hPSC passage number. We also looked at whether hPSC proliferation status affected line differentiation capacity, but found no differences in the distribution pattern of Ki67 during the varying stages of the cell cycle. Most cells were found at a late G1 growth phase and 2-5% of cells were in early G1 or undergoing mitosis (Fig. S2B). We speculated that a poorly maintained line which may not give rise to a SIX1^+^PAX3^+^ population would be poised to differentiate into other cell lineages. To test this, we purposefully differentiated a dCas9-KRAB hiPSC culture containing spontaneously differentiated hPSC colonies and evaluated whether these cells would equally form myogenic progenitors at day 9 compared with well-maintained colonies. We found that this initial pluripotency state prevented cells from differentiating into the myogenic lineage, as demonstrated by the lack of SIX1^+^ expression and abundance of a PAX3^+^PAX6^+^ neuronal cell population (Fig. S2C). These findings provide evidence that hPSC cell fate decisions may be affected by initial hPSC quality.

We also observed differences in media coloration across hPSC lines, indicating changes in pH due to cellular metabolic secretions during directed differentiation (Fig. S3A). Because lactate is a biproduct of cellular metabolic waste, we tested whether lactate levels correlated with pre-myogenic differentiation. Higher levels of lactate corresponded with lower decreases in pH (R^2^=0.94) (Fig. S3B). We found that the dCas9-KRAB line, which had shown the greatest number of SIX1^+^PAX3^+^ cells, also contained the highest concentrations of lactate (11 nM), whereas the less differentiated iPSC75 had a 3-fold lower lactate secretion (3 nM). We also found that cell media consumption throughout the differentiation correlated with the number of cells surviving CHIR treatment (Fig. S3C). Although the seeding density at the start of the differentiation was the same for all hPSC lines, their response to CHIR was dramatically different. At day 2, the KRAB line had reached over 80% confluence, whereas CHIR treatment in the iPSC75 line resulted in poor survival of cell colonies. These results implied that not only does hPSC maintenance affect differentiation, but both seeding density at the start of differentiation and mesoderm induction are crucial regulators for line-line variability.

Notably, we previously used H9 hESC lines to successfully derive myogenic progenitors (Hicks et al., 2018; Xi et al., 2020); however, using RT-qPCR data we demonstrated that the H9 line did not produce SIX1^+^PAX3^+^ cells at day 9 (Fig. 2B). One parameter that differed from previous studies was the source of CHIR. Thus we tested mesoderm induction using CHIR purchased from Stem Cell Technologies (SCT) or Tocris Bioscience at 10 µM and 15 µM, as we observed that the H9 line appeared to be less responsive to 10 µM CHIR from SCT. Not only did we find differences in CHIR-induced cell morphology, but we also found that CHIR potency differed between the two companies. Although 15 µM of Tocris CHIR was toxic and killed cells, the cells treated with 15 µM CHIR from SCT survived to similar levels as 10 µM Tocris CHIR conditions (Fig. S4A). This was tested in side-by-side comparisons using the same initial cell seeding densities. When comparing the gene expression for myogenic and non-myogenic markers induced by 10 µM CHIR from both companies using the H9 and dCas9-KRAB hPSC lines, we discovered that Tocris CHIR induced a 4- and 10-fold increase in expression of *PAX3* and *SIX1*, respectively, than CHIR from SCT in both hPSC lines (Fig. S4B); these results suggest that commercial source of small molecules like CHIR affects myogenic differentiation.

### CHIR and cell seeding density modulate myogenic lineage commitment

**Fig. 3.**
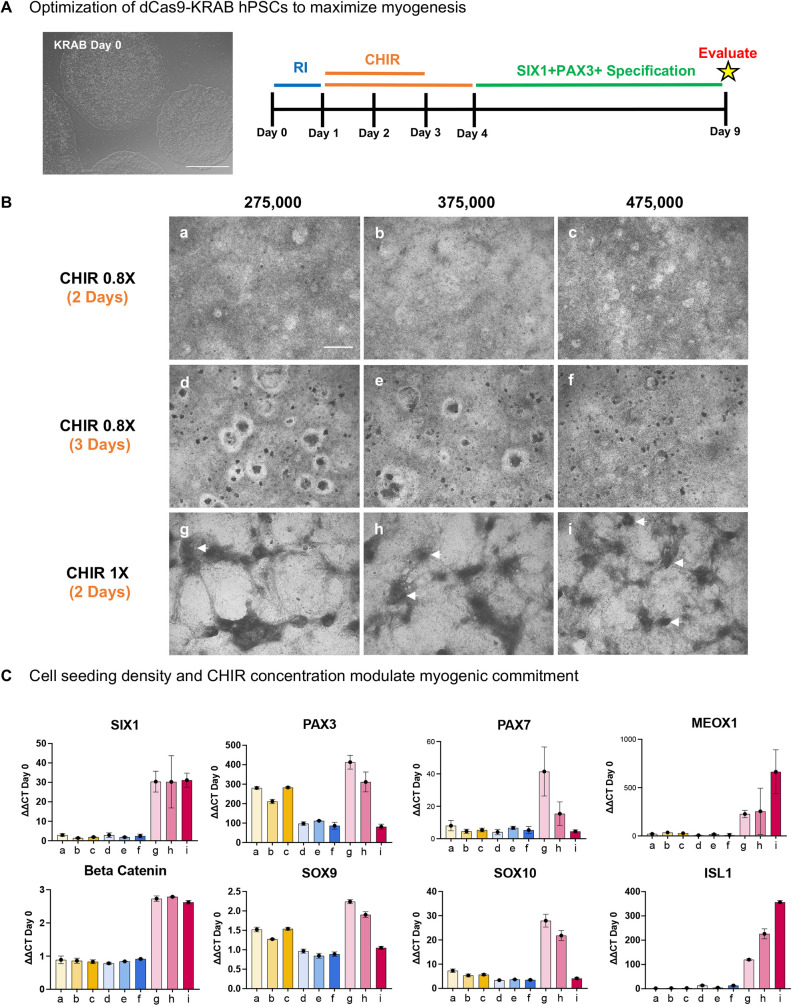
**CHIR sensitivity and cell-cell contact modulate myogenic commitment in hPSCs.** (A) Schematic of directed differentiation timeline of hPSCs to skeletal muscle; cells treated with CHIR for either 2 or 3 days and evaluated at day 9. (B) Representative brightfield images at day 9 of dCas9-KRAB hPSCs treated with varying concentrations and durations of CHIR99021 at multiple densities. Arrows show 3D structures. (C) Day 9 RT-qPCR normalized to *GAPDH* and relative to expression of hPSCs at day 0 (*n*=1 well/condition). Data are mean±s.d. a-i on *x*-axis correspond to subpanels of B. Scale bars: 50 μm (A); 500 μm (B).

To explore the effects of seeding density and CHIR on SIX1 and PAX3 positivity, we tested a range of seeding densities (275,000, 375,000 and 475,000/well) and concentrations of CHIR for either two or three days using WTC11 hiPSCs and analyzed the emergence of pre-myogenic progenitors at day 9 by RT-qPCR (Fig. 3A). We observed dramatic changes in cell morphology largely dependent on the concentration of CHIR but not seeding density. Treatment with higher CHIR concentrations induced formation of distinguishable 3D morphological structures, whereas lower CHIR concentrations produced a flat monolayer of cells (Fig. 3B). By gene expression, higher concentrations of CHIR resulted in increased β-catenin (*CTNNB1*) and *SIX1* regardless of seeding density. *PAX3* and *PAX7* however, were dependent on both seeding density and CHIR concentration, where higher seeding densities resulted in lower *PAX3* and *PAX7* expression. We found that expression of non-myogenic lineages including chondrocytes (*SOX9*) and neural crest cells (*SOX10*) paralleled the expression of *PAX3* and *PAX7* indicating that higher densities were inhibitory of their expression with potentially both non-myogenic cells aiding in the rise of a PAX3, PAX7 myogenic cell. In contrast at higher cell seeding densities, *ISL1* and *MEOX1* expressing cells increased, indicating that higher densities may be enabling the differentiation of cardiac and sclerotome progenitors (Fig. 3C). Here, we demonstrate that fine tuning of seeding density and proper CHIR treatment induces the formation of 3D structures by day 9 and that this morphological phenotype correlates with the highest expression of myogenic-related genes including *SIX1* and *PAX3*.

### *SIX1* inhibition by CRISPRi restricts hPSC myogenic differentiation

To validate that *SIX1* is necessary for efficient myogenic induction, we used CRISPR interference (CRISPRi) via a dCas9-KRAB hPSC line. Comprising a constitutive active promoter driving expression of a deactivated Cas9 fused to a KRAB repressor domain, we were able to conduct reversible inhibition of *SIX1* upon gRNA transfection. To optimize transfection of gene-specific gRNAs, we evaluated several timepoints early during directed differentiation. We found that transfection during early time points enabled GFP expression in 30-50% of cells, whereas GFP transfection was markedly reduced at later time points (Fig. S5). We reasoned that gRNAs, which are much smaller than the pmaxGFP plasmid used for optimization, would have higher transfection efficiencies but may not target all cells, thus we added gRNAs on multiple days to increase efficiency of knockdown.

**Fig. 4.**
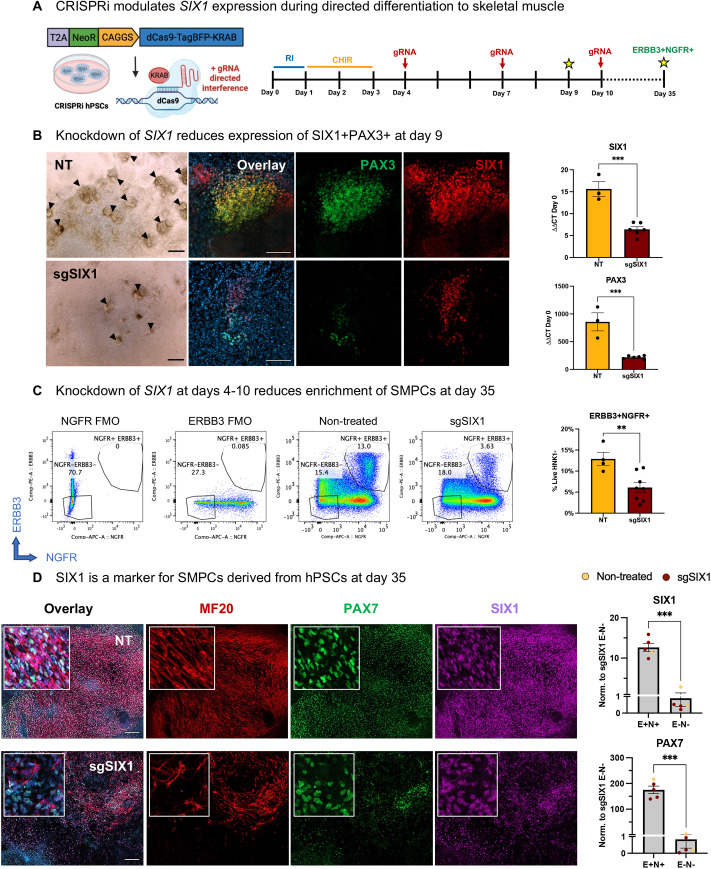
**CRISPR interference modulates SIX1 expression during directed differentiation to muscle.** (A) Schematic of inducible CRISPRi hPSCs and experimental design. sgSIX1 was transfected using Lipofectamine reagent at days 4, 7 and 10 during differentiation and analyzed for expression of SIX1 and PAX3 at day 9. At day 35, cultures were FACS-enriched for ERBB3^+^NGFR^+^ myogenic progenitors. (B) Representative brightfield image at day 9; arrowheads show SIX1^+^PAX3^+^ clusters. Non-treated and gRNA-treated cultures stained with Hoechst 33342 (blue), PAX3 (green) and SIX1 (red) during hPSC-SMPC derivation. RT-qPCR analysis shows relative expression to *GAPDH* and normalized to hPSCs at day 0 (*n*=3-6 biological replicates). ***P<0.001 (two-tailed unpaired *t*-test). (C) At day 35, cultures were sorted for ERBB3^+^NGFR^+^. FACS plots depict percentages of SMPCs from FMO controls, non-treated (NT) and sgSIX1 treatments (*n*=4-6 biological replicates). **P<0.01 (two-tailed unpaired *t*-test). (D) NT and sgSIX1-transfected cultures at day 35 identifying Hoechst 33342 (blue), PAX7 (green), MF20 (red) and SIX1 (magenta). Positive and negative ERBB3 (E) and NGFR (N) FACS-enriched populations were analyzed by RT-qPCR for expression of *PAX7* and *SIX1*. Levels show relative expression to *GAPDH* and normalized to expression of transcripts in the ERBB3^−^NGFR^−^ groups (*n*=4-5 biological replicates). ***P<0.001 (two-tailed unpaired *t*-test). Data are mean±s.e.m. Scale bars: 100 μm (B); 200 μm (D).

We then performed *SIX1* knockdown at days 4, 7 and 10 during directed differentiation of hPSCs to SMPCs and evaluated the cultures for expression of SIX1^+^PAX3^+^ myogenic progenitors at day 9, and differentiation of PAX7^+^ myogenic progenitors at day 35 (Fig. 4A). At day 9, we found a dramatic reduction of the 3D structures in the cultures incubated with *SIX1* gRNAs compared with the non-treated (lipofection only) controls; this resulted in minimal expression of both SIX1 and PAX3. By RT-qPCR, we found that inhibition of *SIX1* not only resulted in a significant 3-fold decrease of *SIX1* expression, but also contributed to a significant 2-fold decrease in *PAX3* (Fig. 4B). We next wanted to determine whether this early inhibition of *SIX1* during skeletal muscle differentiation negatively affected the derivation of ERBB3^+^NGFR^+^ skeletal muscle progenitors later during directed differentiation (day 35). Fluorescence-activated cell sorting (FACS) analysis revealed a significant reduction in the percentage of ERBB3^+^NGFR^+^ progenitors in the sgSIX1 conditions compared with controls (Fig. 4C). These data indicate that *SIX1* may be involved in the regulation of early hPSC commitment to skeletal muscle. Immunofluorescence analysis at day 35 showed a clear reduction in the presence of PAX7 and myosin (MF20) in the sgSIX1 group compared with controls. These results served as an additional confirmation that, in the absence of *SIX1*, hPSC SMPCs differentiate poorly. Further evaluation by RT-qPCR demonstrated that *PAX7* and *SIX1* were significantly upregulated in the ERBB3^+^NGFR^+^ FACS-enriched population compared with the ERBB3^−^NGFR^−^ cells, which confirms that *SIX1* is co-expressed with *PAX7* in SMPCs later in differentiation (Fig. 4D).

### CRISPRi reduces non-myogenic lineages independent of SIX1 expression in hPSCs

During embryogenesis, muscle progenitors derived from the dermomyotome are closely associated with a diverse array of cell types that provide signals for myogenic specification. Although hPSC directed differentiation aims to generate a pure population of skeletal muscle progenitors, there are a multitude of cell lineages that also arise in culture; whether these non-myogenic cells serve to inhibit or stimulate the formation of skeletal muscle *in vitro* remains to be explored. Modifications of CHIR and seeding density also caused concurrent specification of chondrocyte and neural crest cells alongside myogenic cells, whereas opposing conditions favored derivation of cardiac progenitors. To investigate whether these cell types have either an inhibitory or stimulative role during myogenesis *in vitro*, we took advantage of our CRISPRi hPSCs.

**Fig. 5.**
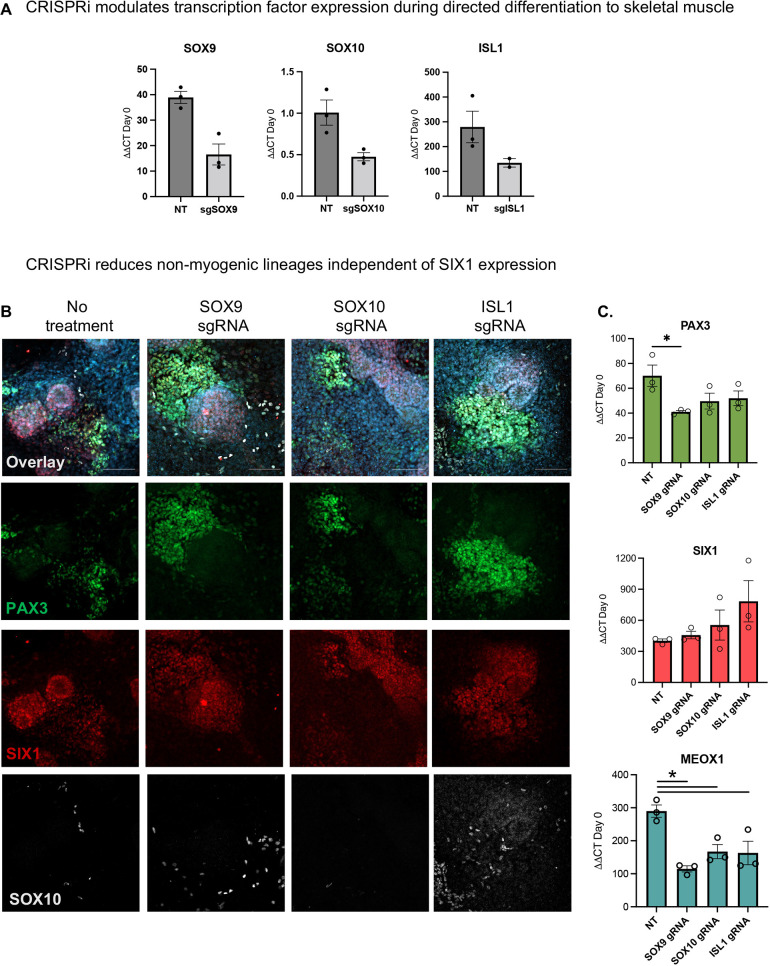
**CRISPR interference of non-myogenic lineages improves myogenesis *in vitro.*** (A) RT-qPCR showing knockdown efficiency levels of gRNA target genes *SOX9*, *SOX10* and *ISL1* at day 9 normalized to *GAPDH* and relative to expression of hPSCs at day 0 (*n*=2-3 biological replicates). NT, not treated. (B) gRNA-treated cultures stained with Hoechst 33342 (blue), PAX3 (green), SIX1 (red) and SOX10 (white) antibodies (left). RT-qPCR showing levels of *PAX3*, *SIX1* and *MEOX1* in gRNA-treated culture conditions at day 9 (right). Levels show relative expression to *GAPDH* and normalized to hPSCs at day zero (*n*=3 biological replicates). *P<0.05 (one-way ANOVA). Data are mean±s.e.m. Scale bars: 100 μm.

To investigate how downregulation of the master regulators for chondrocyte, neural crest and cardiac progenitors affected the genesis of myogenic progenitors *in vitro*, we targeted gRNAs against *SOX9*, *SOX10* and *ISL1* during the directed differentiation and observed 40-60% knockdown of gRNA-targeted gene expression (Fig. 5A). We stained the cultures for these markers and verified knockdown and reduced expression (Fig. S5). We next evaluated the expression of *PAX3*, *SIX1* and *MEOX1* using RT-qPCR (Fig. 5B). All gRNA-treated conditions resulted in a reduction of *MEOX1*, which further indicates that MEOX1 is expressed by multiple cell types*.* We also found that *SOX9* inhibition had the greatest reduction of *PAX3* and *MEOX1* (*P*<0.05) but did not change *SIX1* expression. We found that knockdown of *ISL1*-expressing cells contributed to a 2-fold increase in *SIX1* expression, whereas in all gRNA-treated conditions, the expression of *PAX3* and *MEOX1* (Fig. 5C) was downregulated. This positive shift in *SIX1* expression reveals an interplay between non-myogenic PAX3^+^ cells (SOX10^+^), sclerotome associated cells (MEOX1, SOX9), and ISL1^+^ progenitors, with all cell types potentially competing to expand. These results show that modulating the emergence of non-myogenic cells, specifically developing ISL1 progenitors early during directed differentiation, may upregulate SIX1 to favor the activation of the myogenic program.

### Myogenic differentiation and maturation of early SIX1^+^PAX3^+^ progenitors form PAX7^+^ and myosin^+^ skeletal muscle cells *in vitro*

**Fig. 6.**
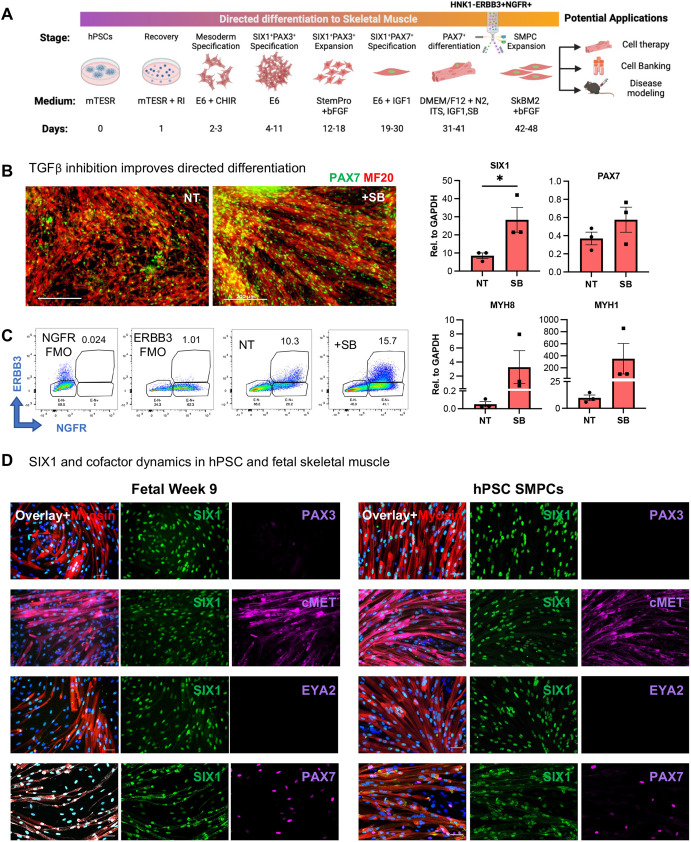
**SIX1 is expressed by hPSCs, fetal PAX7^+^ SMPCs and myotubes and is regulated by TGFβ.** (A) Schematic of complete directed differentiation of hPSCs to skeletal muscle. (B) Cultures at the end of differentiation show PAX7 (green) and MF20 (red) with 5 μM SB431542 treatment (SB) or without treatment (NT). RT-qPCR quantification (right) shows myogenic gene expression±SB relative to *GAPDH* (*n*=3 biological replicates). *P<0.05 (two-tailed unpaired *t*-test). (C) At the end of directed differentiation, cultures were sorted for ERBB3^+^NGFR^+^. FACS plots depict percentages of SMPCs from FMO controls, NT and SB treatments. (D) ERBB3^+^NGFR^+^ SMPCs from human fetal week 9 cells (left) or derived from hPSCs (right) were expanded and differentiated to myotubes (red). Cultures were stained with Hoechst 33342 (blue) and evaluated for SIX1 (green), PAX3, cMET, EYA2 and PAX7 (magenta). Data are mean±s.e.m. Scale bars: 200 µm (B); 50 µm (D).

We have demonstrated that SIX1^+^PAX3^+^ progenitors can be used to screen for the emergence of skeletal muscle progenitors and that, without these, directed differentiation to muscle is inefficient. Improved derivation and enrichment of skeletal muscle progenitors at 5 weeks can lead to several clinical applications (Fig. 6A). Therefore, to further improve skeletal muscle differentiation from hPSCs, we sought to improve myotube maturation. We have previously demonstrated that TGFβ inhibition improves SMPC myotube differentiation (Hicks et al., 2018), so we tested whether inhibition of TGFβ during the final stages of directed differentiation also enhanced myotube differentiation. Multiple studies have revealed that TGFβ inhibition improves the maturation of embryonic myofibers towards a secondary myogenesis, which supports more mature PAX7^+^ SMPCs (Cusella-De Angelis et al., 1994; Massague et al., 1986; Melendez et al., 2021). Thus, we added a potent TGFβ inhibitor (SB431542; SB) during the final week of directed differentiation containing both myotubes and SMPCs, and measured *SIX1*, *PAX7*, *MYH8* and *MYH1* expression. Compared with non-treated cultures, SB-treated myotubes were larger, more organized and expressed increased fetal and adult myosin genes *MYH8* and *MYH1* (Fig. 6B). RT-qPCR analysis found an increase in SMPC genes *SIX1* and *PAX7* with SB treatment, which also resulted in a 75% percentage increase in ERBB3^+^NGFR^+^ SMPCs captured by flow cytometry (Fig. 6C). To determine whether SIX1 was maintained on SMPCs, and whether its co-factors arose later, we performed immunofluorescent staining and used FACS-enriched ERBB3^+^NGFR^+^ human fetal week 9 SMPCs for comparison. We found that SIX1 was expressed by both PAX7^+^ SMPCs and differentiated myosin^+^ myotubes, whereas PAX3 expression was absent at these later time points. We also found that cMET was expressed, but a key reported SIX1 co-factor, EYA2, was not expressed by SIX1^+^ muscle cells (Fig. 6D).

### Transcriptomics across human myogenesis and hPSCs identify distinct SIX1 co-factor and signaling differences

**Fig. 7.**
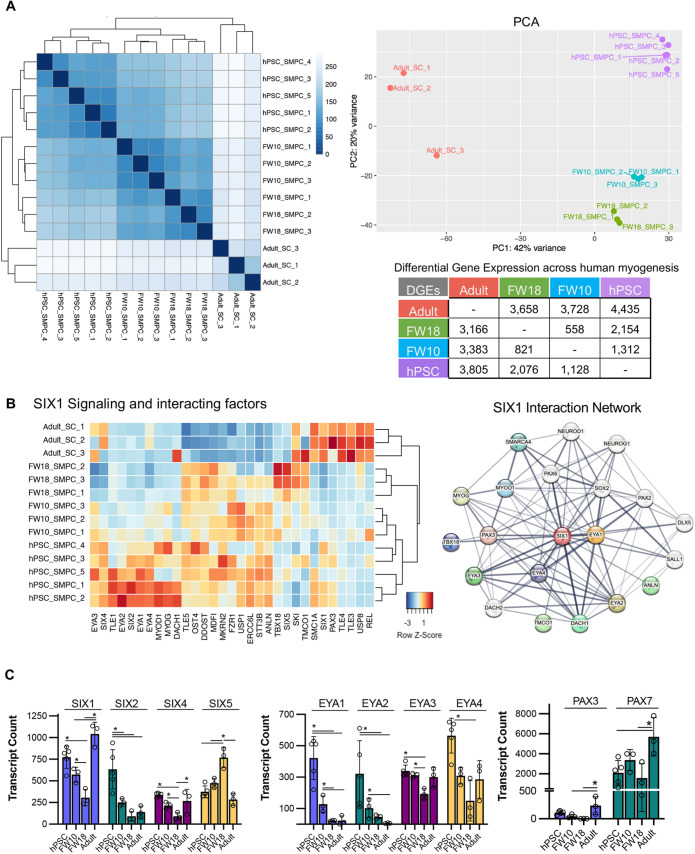
**Signaling and cofactor regulation of SIX1 across myogenic development and in hPSCs.** (A) DGE and PCA across the different samples. Sample-to-sample distance plot as described by DESeq2 from RNA-seq from hPSC, embryonic and fetal SMPCs, and adult SCs (*N*=3-5) (left). Dendrogram shows hierarchical relationship across development (top right). Number of upregulated genes also shown (bottom right). (B) Heatmap of *SIX1* signaling across hPSC, embryonic and fetal SMPCs and adult SCs (left). Functional protein association network of *SIX1* generated by STRING (right). Genes not expressed in our transcriptomic data are shown in white bubbles. (C) RNA-seq transcript counts generated with DESEq2 showing expression of SIX, EYA and PAX genes across samples. *P<0.05 (DESEq2 with FDR<0.05). Data are mean±s.d.

To better understand SIX1 signaling and co-factor activation in human development, we profiled the transcriptomics of FACS-enriched hPSC SMPCs using bulk RNA-seq across five directed differentiations that had been differentiated for 6-7 weeks and optimized using the approaches described throughout this manuscript (Fig. 6A). We included an in-depth comparative analysis on the gene signature of FACS-enriched human embryonic (week 9-10) and fetal (week 16-20) SMPCs, and adult satellite cells (SCs) (years 18-50), which we used to evaluate SIX1 and co-factor signaling dynamics across human myogenesis (*N*=3-5 per group). SMPCs were enriched using the surface markers Lin-ERBB3^+^NGFR^+^ (Hicks et al., 2018), and adult SCs using Lin-CD82^+^NCAM^+^ (Alexander et al., 2016). FACS-enriched populations were immediately collected and processed for RNA-seq analysis to enable a more complete and accurate reconstruction of gene expression levels, and datasets were provided including transcript counts and differential gene expression (DGE) across SMPC and SC myogenesis (Tables S1 and S2). Our RNA-seq analysis revealed thousands of differentially expressed genes across the transcriptome of biological samples (Fig. 7A; Fig. S6). Sample-by-sample and principal component analysis (PCA) determined all biological replicates clustered by biological age. PC1 showed that hPSC and fetal SMPCs clustered with each other; however, PC2 showed that embryonic week 10 and fetal week 18 SMPCs clustered together rather than with hPSC SMPCs. Adult SCs were least similar to SMPCs from both hPSC and fetal skeletal muscle.

Our whole transcriptome datasets can be used for analysis of many transcriptomic pathways, and we used these datasets to evaluate SIX1 signaling in more detail (NCBI GEO GSE234616; Fig. 7B). We used STRING, functional protein association network analysis and conducted a literature search to identify protein associations of SIX1 and then cross referenced these to our RNA-seq datasets to generate the heatmaps and transcriptome counts from DESeq2 data (Table S1). Analogous to the clusters shown on PCA, we found that *SIX1* signaling factors clustered by developmental age and in hPSCs. We found that both *SIX1* and *PAX7* were expressed by all SMPCs/SCs across development, which confirms our immunofluorescence data; however, *SIX1/2* decreased during fetal myogenesis and instead we found high levels of SIX5 at this time point (Fig. 7B,C). We found that cofactors *EYA1/2* were highly expressed by hPSCs and embryonic SMPCs but were not expressed by fetal SMPCs or adult SCs. Alternatively, *EYA3/4* expression was comparatively greater during later stages of myogenesis. We returned to our day 9 hPSC differentiations to further test expression of all SIX and EYA genes and found that *SIX1* and *EYA1* were the highest expressed at early myogenic commitment time points (Fig. S6). We also found that other *SIX1* targets and factors described in the literature or by STRING, such as *PAX6* and *DACH2*, were not specific to myogenic cells (shown in white, Fig. 7B), suggesting unique co-factor synergy with *SIX1* during skeletal muscle development.

Due to the rich nature of our RNA-seq datasets, we further evaluated the differences between *in vitro*-derived and *in vivo* samples (Fig. S6). We compiled heatmaps of biological processes that included extracellular matrix (ECM), cell surface receptors, intracellular signaling and transcription factors and used Venn diagrams to compare similarities and differences across samples. Key genes found in *in vivo* samples included high expression of the transcription factor *MYF5*, whereas hPSC SMPCs expressed *MYF6*. Fetal SMPCs, but not hPSC SMPCs, had pronounced expression of several ECM and ECM-receptor pathways that included many of the collagen genes and laminin-interacting genes, which may suggest that structural and secreted ECM factors have an autologous role supporting *in vivo* SMPCs or contributing to the extracellular structure of the muscle. Adult SCs had a combination of maturation genes such *FOXO1*, unique cell surface receptors such as *EGFR* and *OSMR*, and signaling genes such as *NUMB* and *RHOB*, which highlights that the *in vitro*-derived hPSC SMPCs are not equivalent to the bona-fide adult SCs (Fig. S6; Table S2). Further highlighting this point, we found that the SIX1 co-activators and co-repressors are unique across development and adults and, thus, may be regulating these developmental differences between SMPC/SC states.

## DISCUSSION

Understanding and predicting the quality of hPSCs and their differentiated lineage specific cell types is crucial to the translation of personalized cell therapies. We monitored and achieved defined quality of myogenic differentiation from hPSCs through the validation of myogenic progenitor markers expressed in the developing limb bud at week 7 of human embryos. We reveal an emerging SIX1^+^PAX3^+^ population that can be used as an early key transcriptional checkpoint to identify developing pre-myogenic progenitors. We show that *SIX1* is required for efficient myogenic commitment and that *SIX1* knockdown significantly prevented both SIX1^+^PAX3^+^ progenitors from arising and hindered myotube differentiation. By highlighting potential sources of variation for myogenic lineage commitment, we propose a tailored and reproducible approach using the rise of a SIX1^+^PAX3^+^ population as an early screen for improving later myogenic differentiations from any hPSC line. These include modifying cell seeding density and concentration of CHIR, live cell observations on the formation of 3D structures, monitoring lactate production and regulating the emergence of non-myogenic cell types. We demonstrate that *SIX1* identifies early SMPCs from hPSCs and at fetal stages of development, and that its expression is maintained throughout the differentiation of hPSC SMPCs and in myotubes. During the final stages of directed differentiation, TGFβ inhibition can improve myotube maturation as well as yield improved SMPC enrichment by flow cytometry. We also provide a resource highlighting key similarities and differences between hPSC SMPCs and flow cytometry-enriched human embryonic and fetal SMPCs, and adult SCs via bulk RNA-seq.

Our results show that when SIX1^+^PAX3^+^ precursors are robustly expressed early during directed differentiation, that SIX1^+^PAX7^+^ SMPCs can be later obtained with high consistency. We demonstrate that CRISPR knockdown of *SIX1* diminished PAX3 expression and significantly restricted myotube differentiation. We conclude that *SIX1* is an essential regulator of early hPSC myogenesis, which is consistent with multiple studies showing that Six genes are expressed in myogenesis in mouse embryonic and fetal stages of development (Grifone et al., 2005; Laclef et al., 2003) and have been implicated to be in direct control of the myogenic regulatory factors (Giordani et al., 2007; Lee et al., 2018; Relaix et al., 2013). *SIX1* may serve as a general skeletal muscle lineage marker expressed by PAX7^+^ stem and progenitor cells and differentiated myonuclei in humans (Jensen et al., 2022). However, the activity of co-activators and co-repressors that *SIX1* depends on may change throughout development. During early myogenic commitment from hPSCs, *SIX1* is the first of the SIX genes to be expressed, along with *EYA1.* In contrast, we show that *EYA1/2* decrease during fetal development and are not expressed by adult SCs, rather *EYA3* is highly expressed in adult. *SIX1* may also synergize with other SIX genes such as *SIX2/4/5*, which we find expressed in later fetal myogenesis. Human fetal week 18 SMPCs expressed high level of *SIX5*, which may serve to suppress MYOG during the second trimester of myogenesis (Yajima et al., 2010) – this would align with our data showing higher levels of *MYOG* in embryonic week 10 SMPCs and hPSC SMPCs.

In humans, *SIX1* is first detected during the fourth week of somite development, but by itself can also mark epithelial cells that give rise to lineages such as retinal cells and dorsal root ganglia (Fougerousse et al., 2002). Thus, to identify early myogenic precursors, the combination of SIX1 and PAX3 are required. Future research to identify a unique myogenic transcription factor would enable more in-depth lineage tracing to study the transition of early myogenic progenitors to PAX7^+^ SMPCs and myotubes. *In vivo*, Six1 interacts with its cofactors, Eya1 and Eya2, which are highly expressed in the hypaxial dermomyotome and limb, and expression of cMet is required for proper migration of limb progenitors (Epstein et al., 1996; Heanue et al., 1999). However, unlike the fetal limb, we found that SIX1^+^PAX3^+^ hPSC-derived SMPCs did not express EYA2 or cMET at early differentiation time points. Our RNA-seq and immunofluorescence analysis found that these markers are expressed later in hPSC differentiation, but *PAX3* expression is lost by this time. These data suggest that *SIX1* is an upstream myogenic regulatory factor of *EYA2* and *cMET in vitro*, and there may be additional factors that regulate *SIX1* activity at early myogenic time points.

Evaluating hPSC line-line variability and sufficient knowledge about the cell types that arise following directed differentiation protocols is a prerequisite to the production of large-scale and reproducible clinical grade cells. We provide a method to predict the quality of hPSCs for effective derivation of skeletal muscle early during the derivation protocol by regulating CHIR in defined E6 media. A broad range of CHIR concentrations and durations have been used to derive skeletal muscle lineages from hPSCs (2.5-10 μM and 2-6 days, respectively) (Borchin et al., 2013; Chal et al., 2016; Shelton et al., 2014). We show that hPSC lines with diverse genetic backgrounds have significant differences in sensitivity towards the concentration and source of CHIR, and speculate that addition of more than one small molecule such as LDN193189, DAPT or growth factors used by several derivation approaches will require individual optimizations for each hPSC lines to induce the skeletal muscle lineage (Chal et al., 2016; Choi et al., 2016; Sakai-Takemura et al., 2018).

We further demonstrate that the commercial source of CHIR significantly affects myogenic induction and hPSC-induced toxicity, and others have shown that treatment of hPSCs with even lower concentrations of CHIR (3μM, Miltenyi Biotech) can induce toxicity in cultures (Borchin et al., 2013). These findings emphasize variations in small molecule potency between manufacturers, and a need to remain consistent within derivation protocols. In addition to CHIR sensitivity between hPSC lines, we found that the starting cell seeding density is crucial for the derivation of myogenic cells. Cell survival and resulting cell confluence can regulate cell signaling through cell-cell contact and secretion of metabolites that influence lineage-specific differentiation signals. Our work agrees with other studies which report that the starting cell density affects germ layer specification, differentiation efficiencies and functional phenotypes (Gage et al., 2013; Ghosh et al., 2010; Wilson et al., 2015).

Cell proliferation or differentiation pathways can also be mediated by metabolic changes that influence metabolite demand, production of metabolic enzymes and/or as a result of transcriptional changes in metabolite influx (Agathocleous and Harris, 2013). A study revealed that the specification of the paraxial mesoderm relies on the spatial regulation of glycolytic gradients that further regulate Wnt signaling (Bulusu et al., 2017). To improve real-time monitoring and predictive analysis on myogenic differentiation, we performed metabolism assays which showed that increased cellular lactate secretion greater than 5 nM after 24 h could predict expression levels of SIX1 and PAX3. We found that too great of a pH decrease from basal levels can induce cell death, and this can be mitigated by increasing the volume of media during cell feeds to preserve cell viability. Metabolism plays key roles throughout directed differentiation of hPSCs to SMPCs, and interestingly, gene ontology analysis from our RNA-seq revealed an upregulation of glycolytic and lipid metabolism activity in key enzymes involved in cholesterol biosynthesis, such as glucose-6-phosphate isomerase (*GPI*) and lanosterol synthase (*LSS*), that had low expression in adult SCs.

In line with previous studies, we demonstrate that non-myogenic progenitors arise concurrently during hPSC-directed differentiation to skeletal muscle (Xi et al., 2020) and these cells may support or regulate specification of the myogenic lineage. Mesoderm induction *in vitro* may potentially give rise to distinct embryonic regions with lineage-specific progenitors or may give rise to multipotent progenitors, the cell fates of which may be affected by the spatial and intrinsic signals from neighboring cells. This is the first demonstration that early disruptions to sclerotome, neural crest or mesodermal cell lineages through targeted inhibition of *SOX9*, *SOX10* and *ISL1* during directed differentiation of hPSCs downregulate expression of *PAX3* and/or *MEOX1*, which we found was independent of *SIX1* expression*.* In mouse development, Meox1 cooperatively interacts with Pax3 (Mankoo et al., 2003), which supports our data showing mutual knockdown of these transcription factors. We find that MEOX1 is highly expressed by multiple cell types in our hPSC cultures, which points to MEOX1 as a general lineage marker of somite progeny such as mesenchymal and sclerotome cells that form the axial skeleton (Skuntz et al., 2009). Inducible overexpression of PAX3 in hPSCs activates MEOX1 and requires continued induction to commit to myogenesis (Magli et al., 2013). Our results indicate that directed differentiation of SMPCs from hPSCs can give rise to PAX3^+^ multipotent progenitor cells able to shift cell fates and commit to either neural crest (PAX3^+^SOX10^+^), neural tube (PAX3^+^PAX6^+^), mesenchymal fates (PAX3^+^MEOX1^+^) or myogenic fates (SIX1^+^PAX3^+^). It is possible that SIX1 expression at these early stages induces specification of PAX3^+^ cells to myogenic commitment, as *in vitro* studies provide evidence that Six1 can reprogram fibroblasts to myogenic cells; without Six1, fibroblasts cannot be reprogrammed and, without Pax3, these induced myogenic cells cannot differentiate (Lee et al., 2018). We also observed parallel increases in *CTNNB1* with *SIX1* at day 9, suggesting that *CTNNB1* and *SIX1* expression are required for myogenic commitment from mesoderm progenitors (Petropoulos and Skerjanc, 2002). Determining which of these alternative cell fates is required or inhibitory to induce myogenesis needs further exploration.

Recent studies have revealed that hPSCs hold varying potential to differentiate into specific lineages, but hPSC variability remains a challenge for directed differentiation protocols, which have frequently opted to use specific hPSC lines and thereby defeat the purpose of developing personalized medicine. In this study, we highlight SIX1^+^PAX3^+^ as a marker pair to positively identify muscle cells early in directed differentiation, and we present methods to adapt our protocol for efficient differentiation of myogenic cells across hPSC lines.

## MATERIALS AND METHODS

### Cell culture hPSC lines

All hPSC experiments were performed with Embryonic Stem Cell Research Oversight (ESCRO) Committee approval. HPSCS were grown on Matrigel-coated plates (Corning, #354277) in mTESR medium (Stemcell Technologies).

HPSC lines used in this study include H9 (WA09, National Institutes of Health 0062; Figs 1, 2, 5 and 6, Figs S1, S2, S3, S4); H1 (WA01, National Institutes of Health; Fig. 2); dCas9-KRAB (WTC11, Coriell Institute for Medical Research AICS-0090-391; Figs 2, 3 and 4, Figs S2, S3, S4, S5); iPSC75 (Clone 5, University of California, Irvine, Alzheimer's Disease Research Center Induced Pluripotent Stem Cell Core; Fig. 2, Fig. S3), and AAVS1 RFP (WTC11, Coriell Institute for Medical Research; Fig. 2, Fig. S3).

### Differentiation procedure

hPSCs were cultured on mTESR1 for at least three passages. Before the start of differentiation, hPSCs were pretreated with recovery media (10 μM rock inhibitor in mTESR1) for 45 min and then single cell dissociated and seeded on Matrigel-coated plates at a concentration of 275,000-475,000 cells/well, depending on hPSC line, for differentiation. On day 2, cells were treated with mesoderm differentiation media [8-10 μM CHIR (Tocris Biosciences) in E6 media] for a duration of 2-3 days and then switched to SIX1^+^PAX3^+^ specification media (E6 media). Between days 5 and 9, cells should form 3D structures. Depending on morphology and media coloration, cells were switched to SIX1^+^PAX3^+^ expansion media (StemPro-34+basic fibroblast growth factor) between days 10 and 12 for a duration of 6-8 days. Media was then switched to PAX7 specification media (E6 media supplemented with IGF1) for 7-10 days. HPSC-SMPCs were induced to differentiate using PAX7 differentiation media (DMEM/F12 supplemented with N2 and IGF1 media for 5-7 days) and matured with addition of SB431542 to PAX7 differentiation media before flow cytometry. A detailed protocol on hPSC skeletal muscle differentiation including notes and tips on successfully deriving SMPCs can be found in the supplementary Materials and Methods.

### RT-qPCR

Validation of RT-qPCR primers was conducted by performing primer efficiency curves. When collecting cell pellets for RT-qPCR, it is necessary to filter excess ECM contents using 70-100 µm filters from SMPC-derived cultures, as these tend to clog RNAeasy columns and affect RNA yield quality. Total RNA was collected using Qiagen RNeasy Plus Mini Kits, and RNA yield and quality was analyzed using a NanoDrop 2000 spectrophotometer. cDNA was synthesized using iScript Reverse Transcription Supermix (Bio-Rad), and gene expression was determined using a Quantstudio 7 Flex Real-Time PCR System with SYBR Green PCR Master Mix (Bio-Rad). Experiments were conducted using either 96-well or 384-well plates, with the amounts of SYBR Green, RNAse free water and primers adjusted accordingly. RT-qPCR data was analyzed using ΔΔCT in which every hPSC line compared with its own day 0 pluripotent control. See Table S3 for primers.

### Transfection in hPSCs

Oligos were briefly centrifuged to ensure RNA was collected at the bottom and then rehydrated with the appropriate amount of 1× TE buffer to make a final sgRNA concentration of 100 µM. A total of 3 µg/well of sgSOX9, sgSOX10 and sgISL1 targets (two sgRNA targets per gene; 1.5 µg per sgRNA target) were transfected using Lipofectamine Stem transfection reagent protocol (STEM00001, Invitrogen) at days 3 and 5 during the directed differentiation protocol. A total of 2.6 µg/well of sgSIX1 targets (two sgRNA targets per gene; 1.5 µg per sgRNA target) was transferred using the same gRNA protocol at days 4, 7 and 10 of the directed differentiation protocol. See Table S4 for gRNAs.

### Immunocytochemistry

All cells were fixed with 4% paraformaldehyde (PFA) for 15 mins, and permeabilized using 0.5% Triton X-100 for 15 mins. Fixed cells were blocked in 5% bovine serum albumin with 10% fresh goat serum for 1 h. Cells were incubated overnight at 4°C in primary antibody solution containing fresh 5% goat serum. Following incubation, cultures were washed three times using PBS and followed by secondary antibody incubation containing fresh 5% goat serum for 1 h at room temperature. See Table S5 for antibodies.

### Acquisition and immunohistochemistry of formalin-fixed paraffin-embedded fetal tissue

Embryonic human tissues at week 6-7 were previously acquired for use in Xi et al (2020), and frozen tissue was taken for additional sectioning and staining. Help with embryonic tissue acquisition was originally provided by Katja Schenke-Layland and Simone Liebscher (Eberhard Karls University, Tübingen, Germany). Use of human tissues was Institutional Review Board (IRB) exempt by the University of California, Los Angeles (UCLA) Office of the Human Research Protection Program (IRB #15-000959).

Human tissues were OCT frozen, sectioned and fixed with 4% PFA for 15 mins. Fixed tissues were washed twice in PBS for 5 mins. For removal of non-specific blood vessel staining, this sample preparation protocol was followed: https://transparent-human-embryo.com/?page_id=649&ao_confirm. Further details and modifications on the immunohistochemistry protocol can be found in the supplementary Materials and Methods and Tables S6 and S7.

### Microscopy

Images were taken on a Nikon TIE or Zeiss LSM900 Airyscan microscope. Images were taken per well for quantification. Imaris, Nikon Elements and ImageJ (FIJI) software were used to determined colocalization between markers.

### Lactate assays

During media changes, 1 ml media was collected in 1.5 ml centrifuge tubes and stored at −80°C. We performed 3-6 media collections from independent wells. To measure lactate and pyruvate, we used the Lactate and Pyruvate Assay Kits (MAK064 and MAK071, respectively) from Sigma-Aldrich. Media was centrifuged through a 10kDA column (Vivaspin, 95056) to remove larger proteins, and then a standard curve was generated at 10-2 nM. Condition media was diluted 1:50 in E6 because of saturation. The lactate or pyruvate enzyme was then added for 30 mins and coulometric assays were performed on a plate reader at 570 nm. We found a 0.99 R2 correlation between lactate levels and pH levels across all lines tested. We determined that lactate levels should between 6-10 nM and pH 7.0 after 24 h in culture for efficient differentiations between days 6 and 10 in E6 media.

### Bulk RNA-seq

Five independent hPSC SMPC directed differentiations (*N*=5) and 3-4 biological replicates from embryonic week 9-11, fetal week 17-20 and adult years 25-50 (*N*=10) were dissociated in Collagenase II followed by Collagenase D and Dispase. SMPCs were immediately FACS sorted using ERBB3 and NGFR, and adult SCs using CD82 and CD56. FACS details can be found in the supplementary Materials and Methods. Directed differentiations were performed as described in differentiation procedure in the Materials and Methods. RNA was isolated using RNeasy Microkits (Qiagen). All samples were sequenced using the NovaSeq 6000 which measured 1.6 billion total reads (15-20 million reads/sample). To process FASTQ files, HISATx2 (hierarchical indexing for spliced alignment of transcripts) was used for alignment to the *Homo sapiens* (Hg38) genome (Kim et al., 2015). StringTie used a genome-guided transcriptome assembly (Pertea et al., 2015) and DESeq2 was used for DGE analysis at false discovery rate (FDR) q=0.05 (Love et al., 2014). DESeq uses the Wald Test for determining signficance and uses *P*-value adjusted for multiple testing with the Benjamini-Hochberg procedure, which controls FDR. Venn diagrams were created in Genevenn. DGE between samples and from Venn diagrams were put into NCBI DAVID for functional annotation. Key biological processes and notable genes were reported in figures. Data are provided in Tables S1 and S2. Further details on RNA-seq and analysis can be found in the supplementary Materials and Methods.

## Supplementary Material

10.1242/develop.201509_sup1 Supplementary information
