# Supplementary material for: SIX1+PAX3+ identify a progenitor for myogenic lineage commitment from hPSCs
Source: Development. 2023 Jul 13;150(14):dev201509. doi: 10.1242/dev.201509 (PMC10357017; doi:10.1242/dev.201509)
Supplement: Supplementary information [file develop-150-201509-s1.pdf]

A. SIX1^{+}PAX3^{+} co-expression across protocols

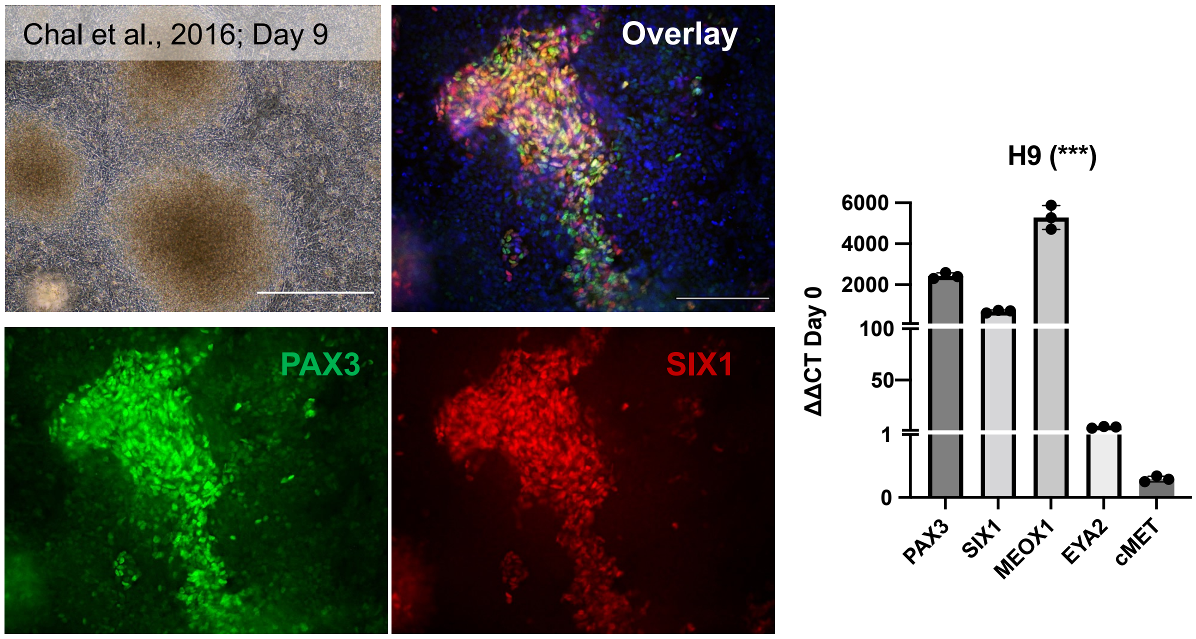

Fig. S1. SIX1+PAX3+ co-expression across protocols

(A) Representative (EVOS XL Core) brightfield image of H9 differentiated cultures at day 9 and immunofluorescent (IF) staining against Hoechst (blue), PAX3 (green) and SIX1 (red) specific antibodies. **Right.** RT-qPCR showing myogenic gene expression at day 9 normalized to *GAPDH* and relative to expression of hPSCs at day zero. (n=3 biological replicates). One way ANOVA resulted in significant difference between groups where (***) represents P value <.001. Data are means ±s.d. Scale bars: 25µm (Brightfield); 100µm (IF).

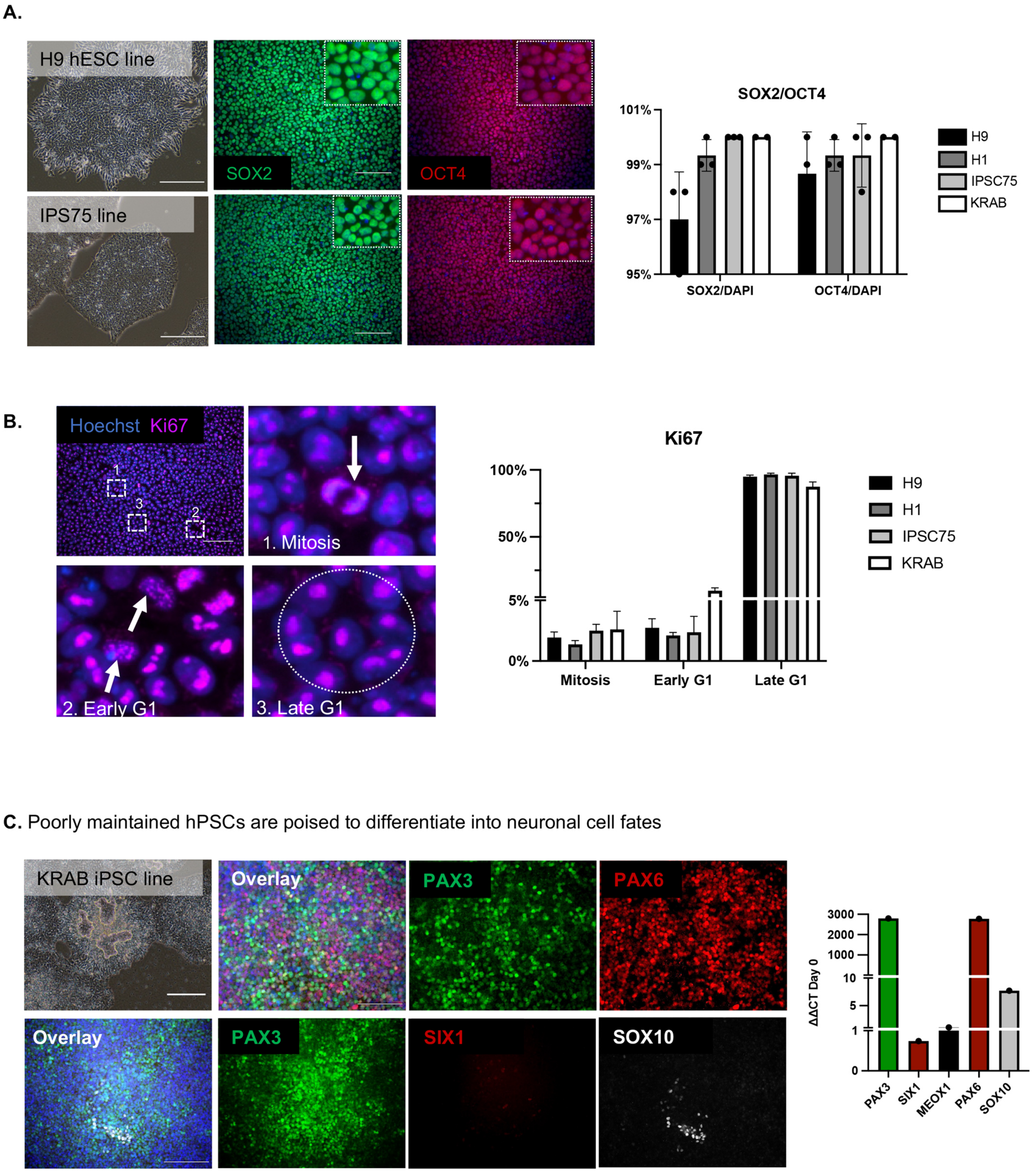
A.
H9 hESC line
IPS75 line
SOX2
OCT4
SOX2/OCT4
101%
99%
97%
95%
SOX2/DAPI
OCT4/DAPI
H9
H1
IPSC75
KRAB
B.
Hoechst Ki67
1. Mitosis
2. Early G1
3. Late G1
Ki67
100%
50%
5%
0%
Mitosis
Early G1
Late G1
H9
H1
IPSC75
KRAB
C. Poorly maintained hPSCs are poised to differentiate into neuronal cell fates
KRAB iPSC line
Overlay
PAX3
PAX6
Overlay
PAX3
SIX1
SOX10
ΔΔCT Day 0
3000
2000
1000
10
5
1
0
PAX3
SIX1
MEOX1
PAX6
SOX10

Fig. S2. Pluripotency and proliferation status does not contribute to hPSC variability during myogenic differentiation.
(A) Representative (EVOS XL Core) brightfield images of H9 hESCs and iPSC75 hPSCs and immunofluorescent staining of day 0 cultures against SOX2 (green) and OCT4 (red) specific antibodies. Scale bars: 10µm (Brightfield); 50µm (IF). **Right.** Quantification showing percentage of hPSC lines expressing SOX2 and OCT4. (1 ROI across n=3 wells). **(B)** Immunofluorescent staining of day 0 cultures using Hoechst (blue) and Ki67 (magenta) specific antibodies. Insets numbered 1-3 provide a zoomed in example of cells undergoing mitosis and early G1 stages (arrows) or cells at late G1 stages (circled). Scale bar: 50µm. **Right.** Quantification showing percentage of varying hPSC lines at either mitosis, early or late G1 stages of the cell cycle. (1 ROI across n=3 wells). **(C)** Representative (EVOS XL Core) brightfield image at day 0 of dCas9-KRAB hPSCs showing differentiated centers and Immunofluorescent staining of day 9 cultures against Hoechst (blue), PAX3 (green), and SIX1 or PAX6 (red), and SOX10 (white) specific antibodies. **Right.** Quantification shows RT-qPCR at day 9 normalized to *GAPDH* and relative to expression of hPSCs at day zero (n=1 well). Data are means ±s.d. Scale bars: 10µm (Brightfield); 100µm (IF).

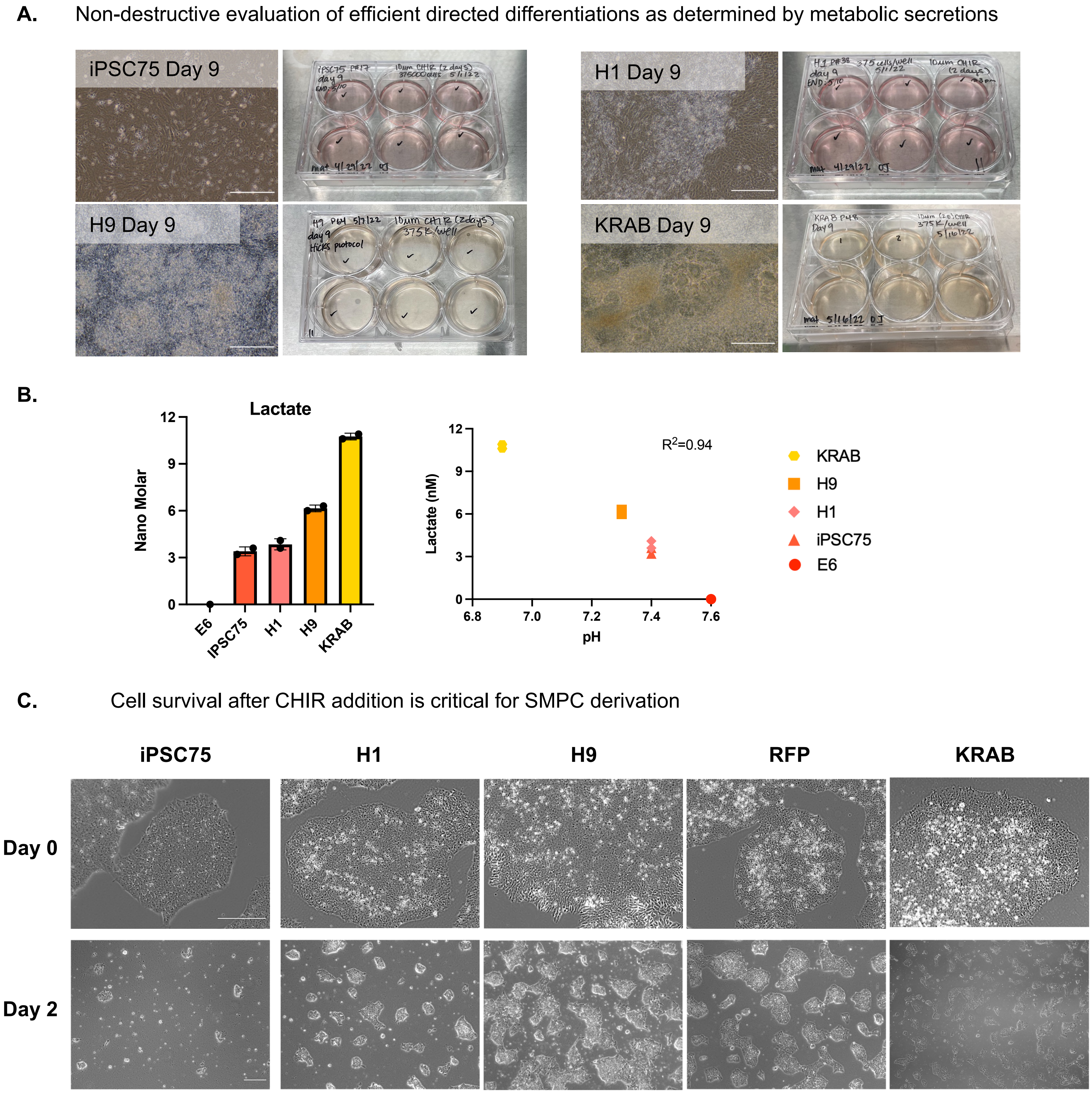

Fig. S3. Metabolic activity corresponds to early myogenic commitment.
(A) Representative (EVOS XL Core) brightfield images of directed differentiation cultures and images to the right show well media coloration at day 9. **(B)** Quantification of hPSC media's lactate concentration (nM) at day 9 normalized to lactate concentrations in E6 media (n=2 biological replicates). Graph correlating pH and lactate concentrations across hPSCs (R^2=0.94). Data are means ±s.d. **(C)** Representative (EVOS XL Core) brightfield images of directed differentiation cultures at day 0 and day 2 after 24hr of CHIR treatment. All scale bars: 10μm.

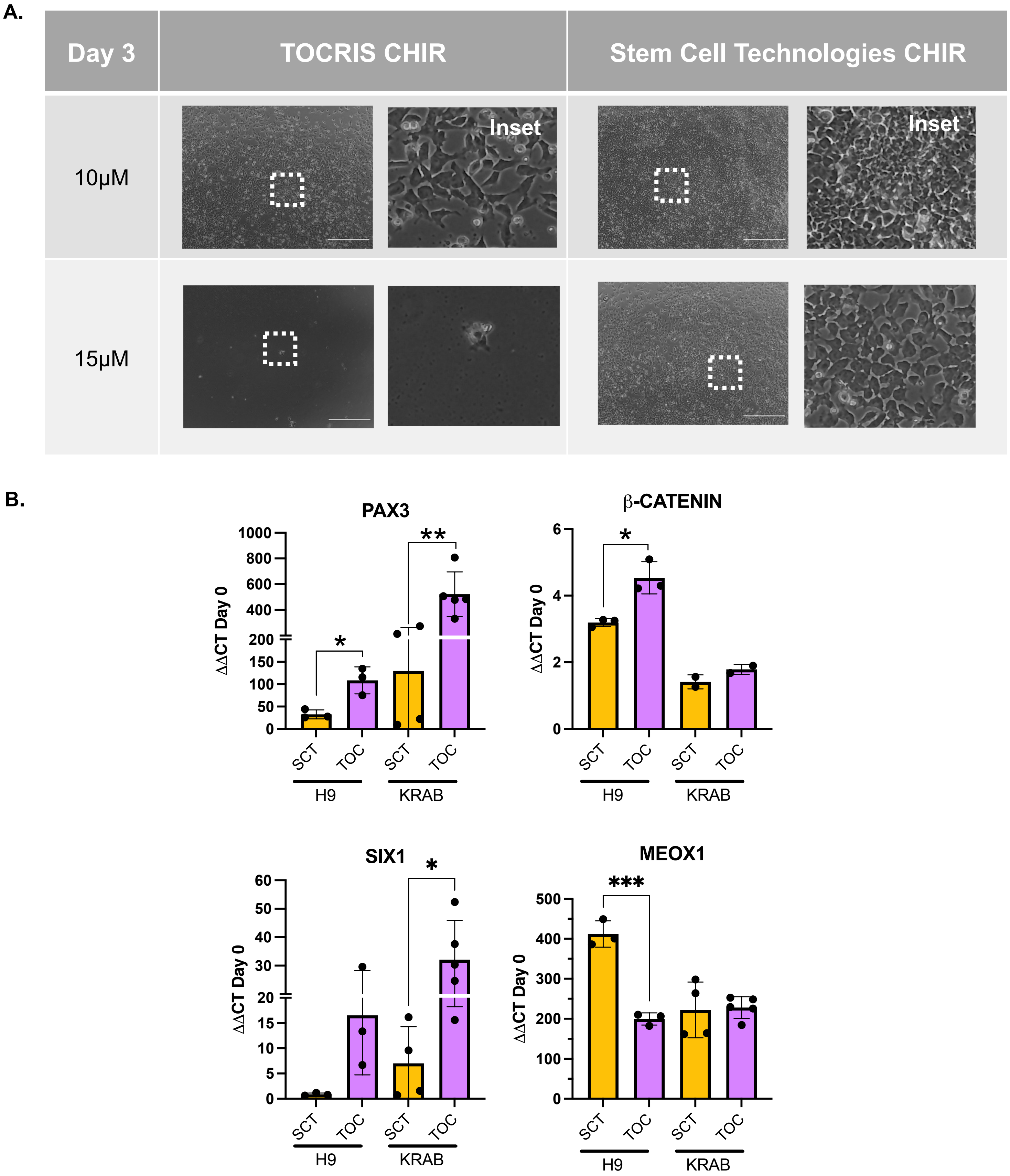

Fig. S4. Commercial source of CHIR regulates early mesoderm induction.
(A) Representative (EVOS XL Core) brightfield images of directed differentiation of dCas9-KRAB cultures at day 3 after two days of CHIR treatment from either Tocris or Stem Cell Technologies at 10μM and 15μM. **(B)** Day 9 RT-qPCR myogenic and non-myogenic gene expression of H9 and dCas9-KRAB cultures treated with 10μM CHIR from either Tocris or Stem Cell Technologies. Expression normalized to *GAPDH* and relative to expression of hPSCs at day zero (n=3-5 biological replicates), t-test analysis shows significant differences between SCT and TOC groups per line. (*) represent P value <0.05, (**) represent P value <0.01, and (***) represent P value < 0.001. Data are means ±s.d. Scale bars: 10μm

A. Lipofectamine optimization during early stages of directed differentiation

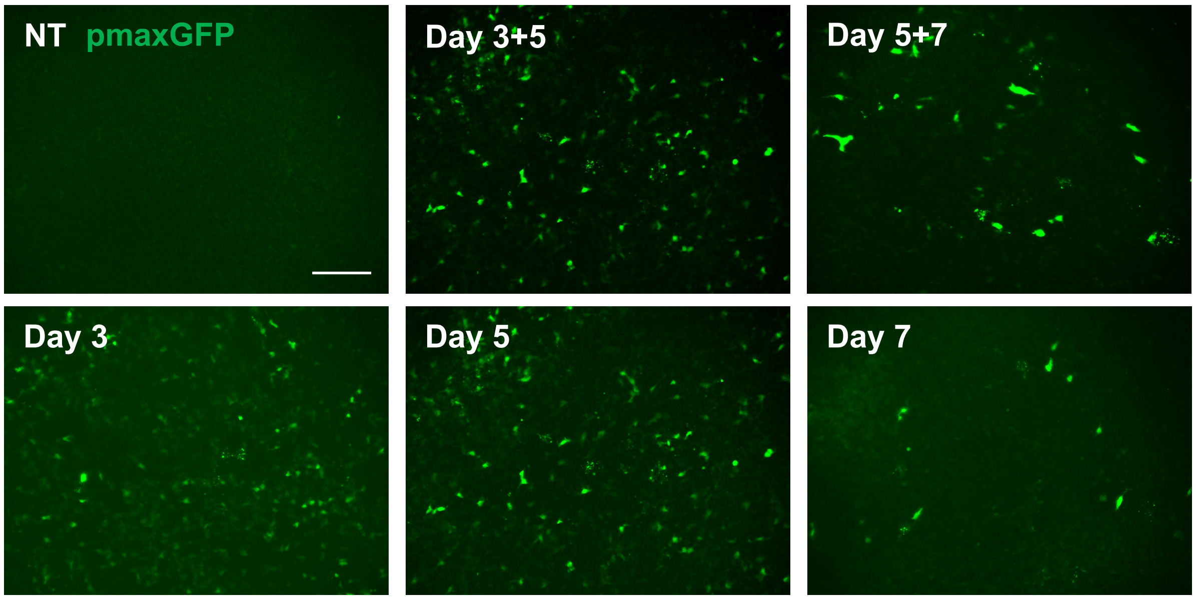

B. Lineage specific knockdown with gRNAs during CRISPR-KRAB hiPSC differentiation

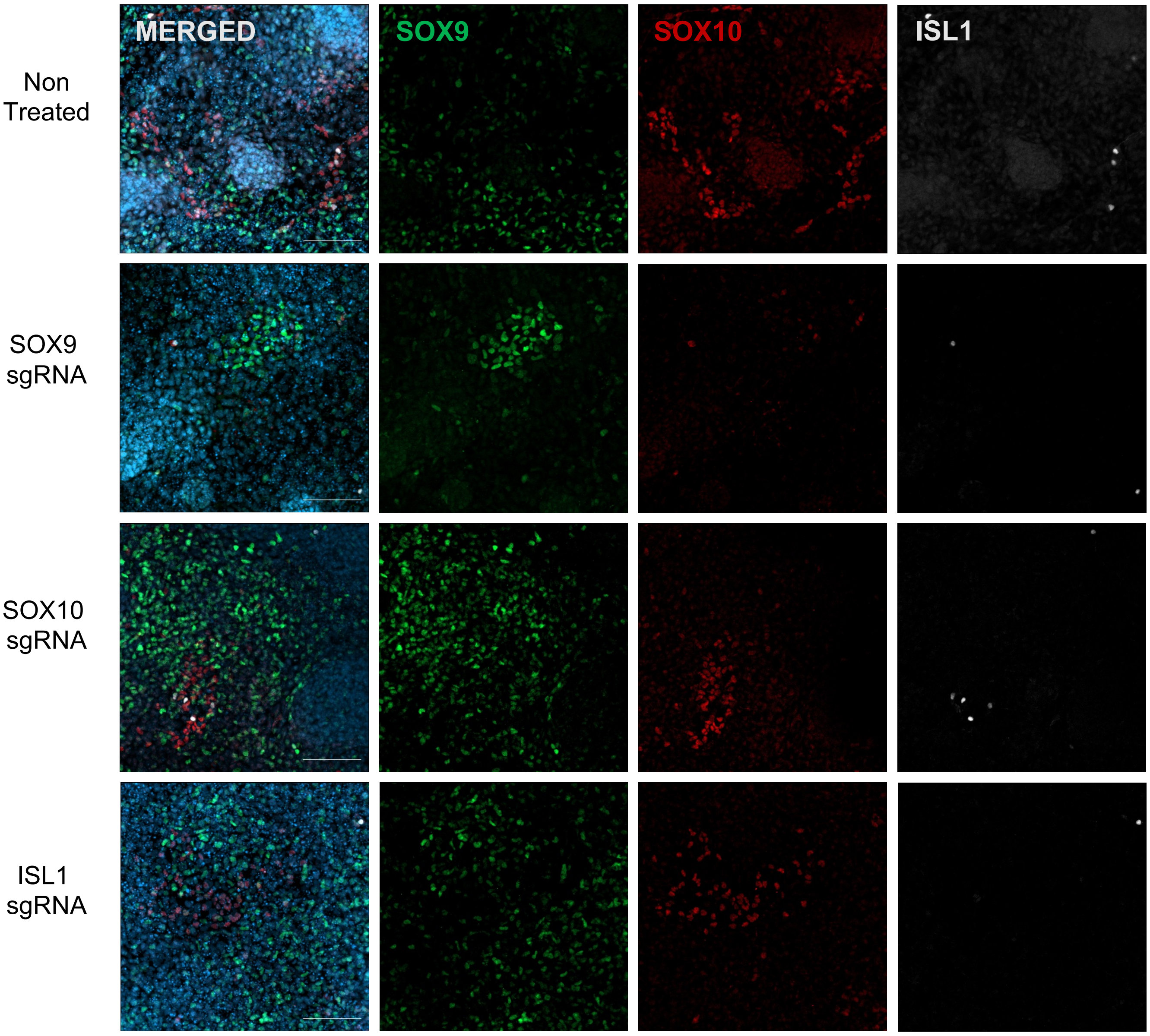

Development • Supplementary information

Fig. S5. Evaluating transfection efficiency during early stages of directed differentiation.
(A) Representative (EVOS M5000) fluorescent images of differentiated dCas9-KRAB hPSCs transfected with pmaxGFP plasmid using LipoSTEM (Thermofisher) at either day 3, day 3 and 5, day 5, day 5 and 7, or day 7 during the directed differentiation compared to non-treated controls. Images were taken 24hrs after lipofection (n=1 well per treatment). **(B)** Representative immunofluorescent images stained for Hoechst (blue), SOX9 (green), SOX10 (red), and ISL1 (white) in gRNA-treated conditions compared to no treatment. Scale bars: 500μm (A); 100μm (B).

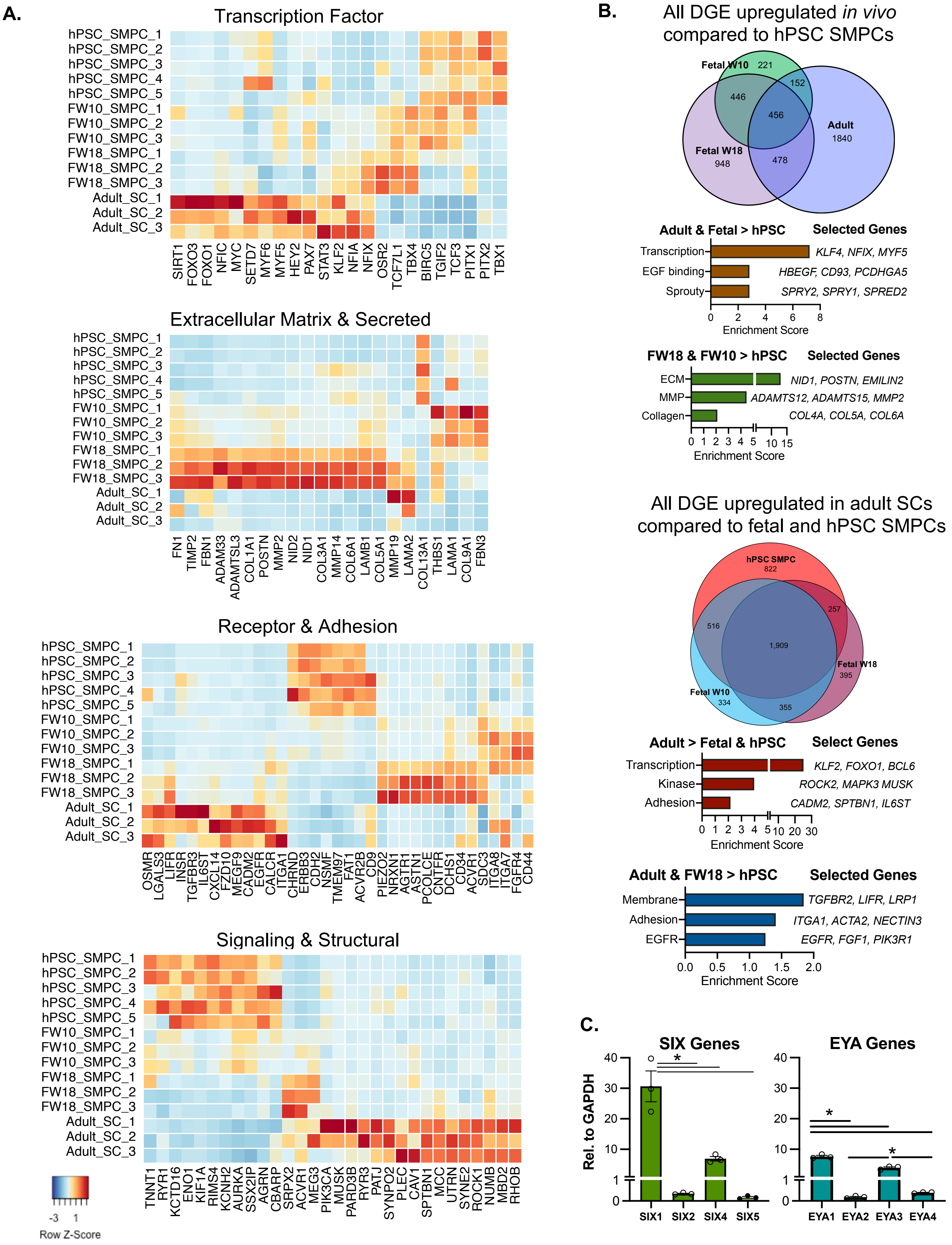

Development • Supplementary information

Fig. S6. RNA sequencing profiling of embryonic, fetal and hPSC SMPCs, and adult SCs. **(A)** Heatmap of selected transcription factors, receptor & adhesion, extracellular matrix & secreted, signaling & structural genes show distinct expression among hPSC, embryonic, and fetal SMPCs and adult SCs. **(B) Top.** Venn Diagram compares differential gene expression between in vivo (derived from primary fetal and adult tissues) and in vitro (derived from hPSCs), and **Bottom.** adult SCs (mature) compared to hPSC and fetal SMPCs (immature). Bar graphs show NCBI DAVID enrichment score from functional annotation of selected pathways related to the SMPC/SC comparisons between groups. **(C)** RT-qPCR showing myogenic gene expression at day 9 normalized to *GAPDH* and relative to expression of hPSCs at day zero. (n=3 biological replicates). One way ANOVA resulted in significant difference between groups where (*) represents P value <.05. Data are means ±s.d.

Development • Supplementary information

Table S1. Transcript Counts of Human Muscle Stem and Progenitor Cells. Buik RNA Seq generated transcript counts using HISAT2, STRINGTIE, and DESeq2. Each column represents a different biological sample, N=3-5. HPSC and fetal SMPCs were FACS sorted for ERBB3 and NGFR, and adult SCs were FACS sorted for CD56 and CD82. Data were used to generate differential gene expression analysis and heatmaps.

Click here to download Table S1

Table S2. Differentially Expressed Genes in Human Skeletal Muscle Stem and Progenitor Cells. DESeq2 generated differential gene expression of biological samples from hPSC, fetal, and adult skeletal muscle. Each tab represents a different comparative analysis. All_[sample] are genes upregulated compared to that sample, and [Sample]_All are genes upregulated in the sample compared to all other samples. Columns showing 1-way analysis are comparing one biological sample to another biological sample, whereas columns showing 2 or 3-way analysis are comparing multiple biological samples and these data were used to generate Venn Diagrams. Row 4 shows the number of differentially expressed genes for each comparison.

Click here to download Table S2

Table S3. Human RT-qPCR Primers

Primer couple sequences used throughout study are listed. Primers were designed in-house using NCBI, primer 3, and Beacon Designer or were taken from published studies. Validation of RT-qPCR primers were conducted by performing primer efficiency curves.

RT-qPCR Primers	Forward (5' → 3')	Reverse (5' → 3')
βCatenin	ATGACTCGAGCTCAGAGGGT	ATTGCACGTGTGGCAAGTTC
cMET	AGCGTCAACAGAGGGACCT	GCAGTGAACCTCCGACTGTATG
EYA2	CACTCCCTGAAGGCACTAAACCTCATC	CTGCATTATCCTCTCGAAGCAGCTCTC
GAPDH	CGCCCCCGGTTTCTATAAATTG	AAGAAGATGCGGCTGACTGT
ISL1	TTTATTGTCGGAAGACTTGCCACTT	TCAAAGACCACCGTACAACCTTTATCT
MEOX1	GGCAGCGTACCCTGACTTC	GGTCCCCATTTCCTTGGAACC
NKX2.5	GTTGTCCGCCTCTGTCTTCT	TCTATCCACGTGCCTACAGC
PAX3	AGCTCGGCGGTGTTTTTATCA	CTGCACAGGATCTTGGAGACG
PAX6	TCTAATCGAAGGGCCAAATG	TGTGAGGGCTGTGTCTGTTC
PAX7	CTGGCCAAAAATGTGAGCCT	TAGGGTTGGGCTGGGAATTG
SIX1	TGTTTGCGCATAAAGGAATG	TGGGAAGGAAAATGCAAAAG
SNAI1	TCGGAAGCCTAACTACAGCGA	AGATGAGCATTGGCAGCGAG
SOX10	CTTCATGGTGTGGGCTCA	CTTGTCACTTTCGTTCAGCAG
SOX2	TCACATGTCCCAGCACTACC	CCCATTTCCCTCGTTTTTCT
SOX9	AGCGAACGCACATCAAGAC	CTGTAGGCGATCTGTTGGG

Table S4. Guide RNA selection for dCas9-KRAB experiments

Sequences for guide used for dCas9-KRAB experiments are provided. We utilized UCSC genome browser, CHOPCHOP, IDT, and Synthego to design gRNAs targeting gene regions against promoter and transcriptional start sites. All sgRNAs were purchased and synthesized by Synthego. Target-specific sgRNAs were prepared per Synthego's synthetic gRNA kit instructions.

**SOX9_1:** UCAGUUUCGAGCUCCGCUUUCGG-modified.
**SOX9_2:** CAGCCAAAGGGCGGACGGUAGGG-modified.
**SOX10_1:** AUUCAGGCUCCGUCCUAACGAGG-modified.
**SOX10_2:** CCUUCCCCAGCCCUCCGCAG-modified.
**ISL1_1:** UUGGUCUGACGCAGCGCGCGGGG-modified.
**ISL1_2:** CUGACCGCUCGGCGAGCCAGCGG-modified.
**SIX1_1:** AAACGACGGCAGCAUCGACA-modified.
**SIX1_2:** AUGUCGAUGCUGCCGUCGUU-modified.
**SIX1_3:** CAAGAACGAGAGCGTACTCA-modified.
**SIX1_4:** TCCTGCGTAAAGCCAAACGA-modified.

Development • Supplementary information

Table S5. Antibody Information:
Primary antibody vendor information and catalog numbers are provided. Dilutions have been optimized for fixed cells or human embryonic tissues.

Primary Antibodies	Vendor	Cat No.	Dilution	IHC with TSA
**C-MET**	Abcam	ab51067	1:100	1:100
**EYA2**	Invitrogen	PA5-66243	1:100	1:200
**ISL1**	DSHB	39.4D5	1:100	
**Ki67**	Proteintech	27309-1	1:1000	
**MEOX1**	Invitrogen	PA596640	1:500	1:1000
**Myosin**	DSHB	MF20-C	1:100	
**OCT4**	Cell signaling	75463	1:200	
**PAX3**	DSHB	Pax3-C	1:200	1:400
**PAX7**	DSHB	Pax7-C	1:30	
**SIX1**	Novus	NBP2-52873	1:100	1:200
**SOX10**	Cell signaling	89356	1:500	
**SOX2**	R&D	MAB2018	1:200	
**SOX9**	Abcam	ab185966	1:200	
**Secondary Antibodies**	**Vendor**	**Cat No.**	**Dilution**	
**mIgG2a-488**	Invitrogen	A21131	1:1000	
**mIgG1-488**	Invitrogen	A21121	1:1000	
**mIgG1-555**	Invitrogen	A21127	1:1000	
**mIgG2b-555**	Invitrogen	A21147	1:1000	
**RbIgG-647**	Invitrogen	A21245	1:1000	

Table S6. IHC protocol Buffers
Preparation for buffers and solutions for immunohistochemistry experiments are listed. For best results, prepare all solutions fresh.

Buffer	Concentrations
**Wash Buffer**	0.05% Tween-20 in PBS
**Blocking Buffer**	3% BSA, 10% GS, 0.05% azide in PBS-T
**Antibody Dilution Buffer**	1% BSA, 10% GS in PBS-T
**HRP Quenching Buffer**	3% H_2O_2 in PBS
**Hoechst**	1:1000 dilution in PBS

Table S7. HRP Antibodies and HRP-based Fluorophores
Secondary HRP antibodies conjugated to HRP used in IHC for signal amplification (tyramide signal amplification) are listed.

HRP Antibodies	Vendor	Catalog Number	Dilution
**Mouse IgG1**	Akoya Biosciences	A10581	1:1000
**Mouse IgG2a**	Akoya Biosciences	A10685	1:1000
**Rabbit IgG1**	Akoya Biosciences	31480	1:1000
**Fluorophore**	**Vendor**	**Catalog Number**	**Dilution in 1X Plus Amplification Buffer**
**Opal 520**	Akoya Biosciences	OP-001001-520	1:200
**Opal 690**	Akoya Biosciences	OP-001006-690	1:200

Supplementary Materials and Methods

Directed differentiation protocol of human pluripotent stem cells (hPSCs) to skeletal muscle progenitor cells (SMPCs)

Culturing hPSC Lines

- ***Note:*** *We have used both mTESR and Essential-8 medium (Gibco, # A151700) with equal efficiency.*
- ***Note:*** *Spontaneously differentiated colonies were manually scraped off using a P200 pipette tip, or by passaging using ReleSR (Stem Cell Technologies # 05872) reagent to selectively detach undifferentiating colonies.*
- ***Note:*** *Quality of hPSCs is essential. Do not start protocol if hPSCs have abnormal growth, J-shaped colonies, are over confluent and are spontaneously differentiating. We find these may lead to increased PAX6 neural progenitors (Figure S2). Some colonies are less behaved.*
- ***Note:*** *Allow hPSC colonies to be passaged at least three times from cryopreservation before starting a differentiation.*
- ***Note:*** *On the final passage prior to starting experiment, hPSC colonies should have been in culture for at least 3 days and have large compact colonies without having over confluence.*

Differentiation procedure

Day 1. hPSC Cell Seeding

Recovery media:

- mTeSR1 medium (Stem Cell Technologies, # 05850)
- 10µM ROCK inhibitor (Stem Cell Technologies, # Y-27632)

1. Allow hPSC colonies to attain 70-80% confluence at the start of differentiation.

2. Pretreat hPSC lines with recovery media for 1 hour prior to starting single cell dissociations.

3. Dissociate hPSC lines in TryPLE for 5-7 minutes. Neutralize 1:3 TryPLE with recovery media and centrifuge at 300g for 5 minutes.

4. Resuspend in 10mL of recovery media, count cells, and seed in Matrigel-coated plates between densities of 275,000-475,000 cells per 6-well plate in recovery media for 1 day (Day 1).

   - ***Note:*** *Performing directed differentiations for the first time with a new hPSC line requires testing multiple seeding densities. Below is a recommendation for seeding densities expressed in cells per well:*
     - *Low seeding density: 45,000 cells (24-well), 250,000 cells (6-well)*
     - *Medium seeding density: 75,000 cells (24-well), 375,000 cells (6-well)*
     - *High seeding density: 125,000 cells (24-well), 650,000 cells (6-well)*

     Total well volume: 0.5ml (24-well), 2ml (6-well)

Mesoderm Induction. Duration 2-3 days

Mesoderm Media:

- E6 media (Thermofisher, # A1516401)
- 10µM CHIR 99021 (Tocris, # 4423)

Development • Supplementary information

5. After 24 hours in recovery media, treat hPSCs with 10µM CHIR99021 in E6 medium for 2 days to induce mesoderm formation. Perform media change daily.

- ***Note:*** *Individual hPSC lines have differing sensitivities to CHIR and should be optimized Similar to Figure 3 of manuscript, we recommend optimizing CHIR concentration for new hPSC lines, depending on CHIR vendor. For Tocris CHIR we recommend testing 5µM, 7.5µM, and 10µM.*
- ***Note:*** *A 3x3 seeding density vs. CHIR concentration matrix can be evaluated at day 9 to select optimal conditions for myogenic differentiation.*
- ***Note:*** *At the completion of CHIR treatment cells should be at 60-70% confluent and have single cell morphology.*

<u>SIX1+PAX3+ Specification. Duration 8-12 days</u>

SIX1+PAX3+ Specification Media:

- Essential 6 media (Thermofisher, # A1516401)

6. E6 medium alone was used to generate mesodermal SIX1+PAX3+ cells for 7-10 days. Perform media change daily.

- ***Note:*** *after the first day in E6 medium cells should reach close to 100% confluence*
- ***Note:*** *3D structures should begin to form within 5-9 days in E6 medium. If these structures have not formed by day 10, consider starting over and either increase cell seeding density or CHIR concentration.* ***Please note that not all 3D structures are created equal****. Look for those shown in* ***A*** *below. These are well anchored to the plate and are free of extracellular matrix attachments; these show positivity for SIX1+PAX3+.The 3D structures shown in* ***B*** *appear to produce excess of extracellular matrix (ECM) to stay attached; these are the worst and will dislodge later in the protocol. If these appear, try increasing either seeding density or CHIR concentration. Successful derivation of skeletal muscle cultures involves 3D structures that are minimally attached to ECM. ECM obstructs skeletal muscle differentiation.*

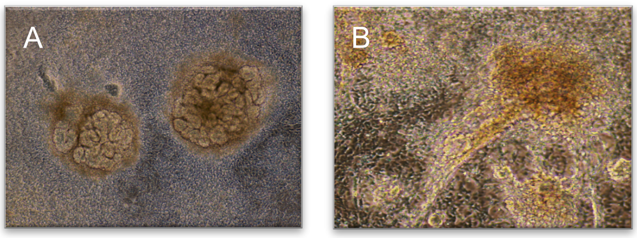

- ***Note:*** *Like Figure S2, yellow coloration of media is a good indication of myogenic differentiation. 24 hours after media change, pH levels should be ~6.9-7.1. pH levels below 7.0 should be increased from 2ml to 2.5ml E6 media to prevent cell death. (Fig.S3).*
- ***Note: Selection of best myogenic conditions using 3x3 matrix:*** *Myogenic differentiation can be evaluated by expression of SIX1, PAX3, and β-Catenin expression. While MEOX1 and ISL1 will be expressed, conditions with the highest expression of MEOX1 and ISL1 should be avoided as these are typically indicative of non-myogenic mesenchymal or cardiac lineages.*

Development • Supplementary information

SIX1+PAX3+ Expansion. Duration 6-8 days.

SIX1+PAX3+ Expansion Media:

- StemPro-34 media (Life Technologies, Cat. 10640-019)
- StemPro-34 Nutrient Supplement
- 5ng/ml bFGF
- 2mM L-Glutamine
- 0.45mM Monothioglycerol
- 11 µg/ml Transferrin

7. Perform media change daily.

PAX7 Specification. Duration 10-12 days.

PAX7 Specification Media:

- E6 Media (Thermofisher, Cat. A1516401)
- Day 23-30, add 10ng/ml IGF1.

8. Perform media change daily.
    - ***Note:*** *Myotube formation can be observed at the end of PAX7 specification.*

PAX7 Differentiation. Duration 5-7 days.

PAX7 Differentiation Media:

- DMEM/F12 (Life technologies, Cat.11330032)
- 1.2% N2 media supplement
- 1.0% Insulin Transferrin Selenium supplement
- 10ng/ml IGF

PAX7 Differentiation with maturation. Duration >5 days

- Add 5µM SB-431542

9. Perform media change daily.
    - ***Note:*** *Spontaneous contractions by skeletal muscle cells can be observed.*
    - ***Note:*** *Skeletal muscle progenitor cells can be enriched at any point after day 42 with similar efficiency through day 55. Longer culturing times may result in cell detachment.*

Development • Supplementary information

Flow Cytometry/FACS Staining Procedure for ERBB3+NGFR+ SMPC Enrichment

FACS Buffer:
2% FBS, 0.5% Pen/Strep in sterile PBS.

Collection Buffer:
MyoCult-SF Expansion Supplement Kit (Stem Cell Technologies, # 05980)
or SkGM-2 Bullet kit (Lonza, # CC-3245) + 10ng/ml bFGF.

Note: Use ~1ml for every 250,000 SMPCs sorted.

Note: We recommend sorting into 5ml FACS tubes which can collect up to 1 million SMPCs per tube.

1. Wash cells with PBS and dissociate hPSC derived SMPCs in TryPLE for 5-7 minutes. Neutralize 1:3 TryPLE with DMEM/F12.
   a. ***Optional**: depending on the 3D structure size/number and ECM deposition by hPSC SMPCs, add 200uL of Collagenase IV (1ug/ml in DMEM) to dissociate. Note: Use a p1000 to gently triturate the cells from the plate.*
   b. ***Optional**: If cells are very clumpy incubate in TryPLE for up to 10 mins*
   c. ***Optional**: Use sterile scissors to cut the ends off the p1000 tips to increase the pipette tip size for easier trituration.*

2. Filter cells to remove ECM and 3D structures through a 100µm filter and verify cells are removed from well plates using microscope.

3. Centrifuge 50ml tube containing cells for 5 min at 300g.

4. Aspirate TryPLE /supernatant and resuspend in FACS buffer.
   a. ***Note**: FACS buffer volume depends on expected cell number, resuspend at expected cell number of 5 million cells per 1 ml.*

5. Count cells.
   a. ***Note:** We prefer to use a hemocytometer over an automated cell counter to better account for cells stuck together. Stay consistent with counting method across users.*
   b. ***Note:** Expect ~2.5 million cells from one 6-well (15 million per 6-well plate) or ~0.6 million cells from one 24-well*

6. Add 2 µL per 1 million cells of Human Fc Block antibody to block non-specific binding of Fc receptors.
7. Divide up samples and controls for staining into multiple 1.5 mL Eppendorf tubes and keep samples > 2 million in 50ml tube.
   a. ***Note:** For samples with cell number 100,000-250,000 dilute FACS buffer to a final volume of 50ul FACS buffer.*
   b. ***Note:** For samples with cell number >2 million dilute FACS buffer to 5 million cells / ml*

8. Add 2 µL per 1 million cells of ERBB3, NGFR, HNK1 flow cytometry antibodies.
   a. ***Note:** when using RFP or BFP hPSCs lines; thaw additional cells that are not transgenic to serve as a negative compensation control.*

9. Include live/dead viability dye (Thermofisher, L34994)
   a. ***Note:** we typically reconstitute dye in 100 µL DMSO and add 0.5 µL per 1 million cells. Avoid repeated freeze/thaw cycles of dye.*

Development • Supplementary information

10. Wash antibodies using 10 parts volume of FACS buffer. Centrifuge cells at 500g for 5 minutes at 12C.

11. Aspirate and resuspend in 1ml FACS buffer per ~5 million cells.

12. Filter cells through 40 µm filter on 5ml FACS tubes.

13. Proceed to FACS sorting using i.e., BD ARIA II.

Culturing SMPCs post FACS

1. Centrifuge FACS tubes for 15 seconds at 300g to collect any cell droplets stuck on the sides of tube. Plate cells at a high density (~250K cells/6-well plate or ~100K in a 24-well plate) onto Matrigel-coated plates.

    Note: *To increase cell yield and reduce further cell stress, we typically do not centrifuge after FACS sorting. Rather SMPCs are directly plated in expansion media containing FACS buffer, which is then removed after 24 hours in culture. FACS buffer should not be more than 20% of total volume.*

PAX7 SMPC Expansion
Expansion Media:
MyoCult-SF Expansion Supplement Kit (Stem Cell Technologies, Cat. 05980)
or SkGM-2 (Lonza, Cat. CC-3244 & CC-3246) + 10ng/ml bFGF

Note*: Do not allow cells to get >90% confluent as the cells can quickly turn on MYOG. Cells can be passaged and be cryopreserved in CryoStor (Biolife Solutions, # 210373). Cells will retain expression of PAX7 and fuse to form myotubes* in vitro *for at least 6 passages.*
Note: *SMPCs can be passaged using 3-4 minutes of TryPLE treatment.*

Optional: PAX7 SMPC differentiation to Myotubes. Duration 5-7 days

PAX7 Differentiation Media:

- DMEM/F12 (Life technologies, Cat.11330032)
- 1.2% N2 media supplement
- 1.0% Insulin Transferrin Selenium supplement
- 10ng/ml IGF
- Day 35-42, add 5µM SB-431542

2. Seed SMPCs on Matrigel-coated plates and allow cells to expand to 80% confluence in expansion media. Once confluent, switch to PAX7 differentiation media. Cells should start differentiating within 3-5 days, continue differentiation through day 7.
    - ***Note:*** *Observe cells daily; myotubes will begin to detach between days 4-7 if additional measures are not taken.*

Acquisition and Immunohistochemistry of Formalin-Fixed Paraffin Embedded Fetal Tissue

Human tissues were OCT frozen, sectioned, and fixed with 4% PFA for 15 minutes. Fixed tissues were washed in PBS 2X for 5 minutes. To remove non-specific blood vessel staining, follow with the following timing modifications:

1. Make all solutions fresh
2. Let tissue come to RT from -20C for about 5 minutes
3. Make a 10% citrate buffer (Sigma-Aldrich, Cat No. C9999) solution in dH_2O and let it come to room temperature

Bleaching at RT

1. Using a slide chamber or Coplin jar, immerse in the following solutions for 20 minutes
    a. 50% methanol in PBS
    b. 80% methanol in PBS
    c. 100% methanol
2. Transfer to a solution of methanol with 6% H_2O_2 and incubate for 1h in the dark at RT

Rehydration

1. Transfer slides and immerse in the following solutions for 20 minutes:
    a. 100% methanol
    b. 80% methanol in PBS
    c. 50% methanol in PBS

Antigen Retrieval

1. Heat citrate buffer solution to 95C
2. Place slides in solution and transfer jar into a pressure cooker with a closed lid for 25 minutes
    a. Make sure there is dH_2O in pressure cooker to create a humid environment.
3. Take jar out from pressure cooker and transfer slides to a Coplin jar filled with dH_2O for 10 minutes to cool

Permeabilization and Blocking

1. Dry most of slide with kimwipe but make sure tissue is still damp.
2. Draw a barrier around tissue with Pap Pen
3. Add wash buffer for 5 mins.
4. Permeabilize tissue with 0.5% Triton-X in PBS for 10 minutes
5. Wash tissue with wash buffer for 5 mins
6. Add 10% goat serum to blocking buffer and block tissue at RT for 1 hour

Antibody Staining

7. Incubate with primary antibody in dilution buffer at 4C overnight
8. Wash with wash buffer briefly
9. Incubate with HRP quenching buffer for 10 minutes
10. Wash with wash buffer briefly once and then wash 3 x 5 mins
11. Incubate with HRP-conjugated secondary antibody in dilution buffer for 45 minutes at RT
12. Wash with wash buffer briefly, wash for 3 x 5 mins, and wash briefly 2X

Development • Supplementary information

13. Incubate with fluorophore-specific TSA working solution in 1X Amplification Diluent (Akoya Biosciences, Cat No. FP 1498) at RT for 10 minutes in dark. All steps from now on should be light protected
14. Wash with wash buffer briefly then wash 3 x 5 minutes
15. If doing multiplexed staining:
    a. Each HRP-conjugated secondary antibody must be applied during separate rounds
    b. Repeat steps 9-14 and proceed to step 16 after last round of secondary incubation
16. Hoechst incubation for 5 minutes
17. Wash with PBS for 5 minutes
    a. ***Optional**: briefly immerse slides in dH_2O in Coplin Jar if glass is dirty prior to cover slipping*
18. Drop mounting medium (Vectashield with DAPI) onto tissues and coverslip. Allow to dry in the dark

Bulk RNA-Seq

Five independent hPSC SMPC directed differentiations (N=5) and 3-4 biological replicates from embryonic week 9-11, fetal week 17-20, and adult years 25-50 (N=10) were dissociated and immediately FACS sorted for SMPC, or SC markers described above. Directed differentiations were performed as described in differentiation procedure in the methods. The cells were collected in SkBM-2, pelleted at 500g, and immediately frozen at -80C. RNA was isolated using RNeasy Microkits (Qiagen) and RNA immediately taken to the Technology Center for Genomics & Bioinformatics (TCGB) at UCLA for bioanalysis and sequencing. Bioanalysis was performed using a D1000 ScreenTape which found hPSC and fetal SMPCs had high RNA yield and quality (RIN >9) allowing for amplification using a Universal Plus mRNA-Seq Library Preparation Kit (Nugen). Adult SCs had lower starting yield and were amplified by Ribo-Zero Plus rRNA Depletion Kit (Illumina). All samples were sequenced using the NovaSeq 6000 which measured 1.6 billion total reads (15-20 million reads / sample).

FASTQ files were then processed using Galaxy (usegalaxy.org). After grooming FASTQ files, HISATx2 (hierarchical indexing for spliced alignment of transcripts), was used to perform a two-pass hierarchical indexing strategy for sensitive spliced alignment of RNA sequencing data to the Homo Sapiens (Hg38) genome (Kim et al., 2015). StringTie used a genome-guided transcriptome assembly approach along with *de novo* genome assembly to improve transcript assembly and estimate expression levels (Pertea

Development • Supplementary information

et al., 2015). To calculate differential gene expression (DGE) and principle component analysis (PCA), DESeq2 used the median count value of all genes from all samples, and then applied a spread function to measure each gene between samples (Love et al., 2014). We evaluated DESeq2 count data and DGE with or without rRNA depleted adult SCs, and found greater than 98% similarity. DESeq2 was used for DGE analysis at false discovery rate (FDR) q=0.05. For stringency we removed rRNA genes, lowly-expressed genes <20 counts, and fold-change <1.5 from DGE analysis. To create Venn Diagrams, we inserted DGEs into Genevenn to find relations between groups and used the ggvenn R package for illustration. DGE between samples and from Venn Diagrams were input into NCBI DAVID for functional annotation. Key biological processes and notable genes were reported in figures. Data are provided in Supplemental Tables S1 and S2. Table S1 show the StringTie and DESeq2 transcriptomic counts of all samples. Table S2 shows the differential gene expression of all samples compared to eachother as denoted by 1-way analysis or used in the Venn diagrams to compare multiple groups in 2-way or 3-way analysis. The values in Row 4 are the number of differentially expressed genes. Each tab of Table S2 represents a comparison to a different biological sample where [Sample]_All refers genes upregulated in a particular biological sample compared to all other groups, and All_[Sample] refers to genes downregulated in a biological sample compared to all other groups.

Development • Supplementary information
