# Supplementary material for: SIX1+PAX3+ identify a progenitor for myogenic lineage commitment from hPSCs
Source: Development. 2023 Jul 13;150(14):dev201509. doi: 10.1242/dev.201509 (PMC10357017; doi:10.1242/dev.201509)
Supplement: Supplementary file 1 [file develop-150-201509_review_history.pdf]

SIX1+PAX3+ identify a progenitor for myogenic lineage commitment from hPSCs

Olga G. Jaime, Jessica Arias, Shreya Pavani, April D. Pyle and Michael R. Hicks
DOI: 10.1242/dev.201509

Editor: Benoit Bruneau

Review timeline

Original submission:	2 December 2022
Editorial decision:	13 January 2023
First revision received:	10 May 2023
Editorial decision:	31 May 2023
Second revision received:	14 June 2023
Accepted:	15 June 2023

Original submission

First decision letter

MS ID#: DEVELOP/2022/201509

MS TITLE: SIX1+PAX3+ Identify a progenitor for myogenic lineage commitment from hPSCs

AUTHORS: Olga G. Jaime, Jessica Arias, April D. Pyle, and Michael R. Hicks

I have now received all the referees' reports on the above manuscript, and have reached a decision. The referees' comments are appended below, or you can access them online: please go to BenchPress and click on the 'Manuscripts with Decisions' queue in the Author Area.

As you will see, the referees express considerable interest in your work, but have some significant criticisms and recommend a substantial revision of your manuscript before we can consider publication. If you are able to revise the manuscript along the lines suggested, which may involve further experiments, I will be happy receive a revised version of the manuscript. Your revised paper will be re-reviewed by one or more of the original referees, and acceptance of your manuscript will depend on your addressing satisfactorily the reviewers' major concerns. Please also note that Development will normally permit only one round of major revision. If it would be helpful, you are welcome to contact us to discuss your revision in greater detail. Please send us a point-by-point response indicating your plans for addressing the referee’s comments, and we will look over this and provide further guidance.

Please attend to all of the reviewers' comments and ensure that you clearly highlight all changes made in the revised manuscript. Please avoid using 'Tracked changes' in Word files as these are lost in PDF conversion. I should be grateful if you would also provide a point-by-point response detailing how you have dealt with the points raised by the reviewers in the 'Response to Reviewers' box. If you do not agree with any of their criticisms or suggestions please explain clearly why this is so.

© 2023. Published by The Company of Biologists under the terms of the Creative Commons Attribution License (https://creativecommons.org/licenses/by/4.0/).

Reviewer 1

Advance summary and potential significance to field

The work of O.G. Jaime et al extends previous published works, by describing a detailed protocol for the culture of human pluripotent stem cells (hPSC) to induce their myogenic directed differentiation. The protocol is well detailed, and many steps are illustrated by the expression of Pax3, a gene suspected to drive the myogenic fate acquisition (activation of Myogenic regulatory factors) of these cultured cells, as already shown in vivo during mouse embryonic development, and by the expression of the myogenic stem cell marker Pax7. During this study the authors show at the RNA and protein-immunochemistry-imaging level that Pax3 and Six1 are coexpressed before Pax7 in cultured cells and represent the first emerging myogenic population. They study the reproducibility of their induction protocol in five distinct hPSC. They also show that application of CHIR to their culture can lead to distinct phenotype, depending of the manufacturer (SCT or Tocris). They further show that the culture medium although favoring myogenic fate acquisition, other cell types are present in their culture, among which Isl1+ cells, Sox9+ cells and Sox10+ cells. By reducing their expression by dCas9-KRAB the authors show by qPCR experiments that somehow they favor the myogenic fate acquisition of their cultured hPSC. Results of bulk RNAseq experiments are presented at distinct times points of the kinetic and compared with embryonic, fetal and adult myogenic stem cells RNAseq.

Comments for the author

In light of the results presented, it would be interesting to discuss the manuscript (DOI: 10.1016/j.nmd.2022.04.006) showing that adult human SC express Pax7 and Six1 (or another manuscript showing coexpression of Six1/Pax7 in human myogenic stem cells), as well as the manuscript of Lee et al (DOI: 10.1038/s41419-018-1114-8) demonstrating that Pax3, Six1 and Eya1 can with Esrrb reprogram fibroblasts into a Pax7 myogenic progenitor cell fate. The authors must better explain the link between their first results describing the best culture ways to get robust PAX3+SIX1+ myogenic progenitors from hPSCs and the transcriptomic data generated. How have the cells been treated to get the hPSC-SMPC1-5 used for the transcriptomic experiments?

In the Figure 6, replace in the transcription factors heat map SPRY1, 4 and Jak1 that are not transcription factors (and place them correctly in the Signaling heat map) by Eya2, Eya3 and Eya4 that may participate with Six1 to the myogenic progenitor fate of the induced hPSC. It would be also interesting due to the role of Eya1 in mouse embryonic development (be careful you wrote line 123 that mouse Eya2 mutant show down regulation of Pax3, but it is the double Eya1/Eya2 mutant that presents this phenotype in Grifone et al, 2007, the single Eya2 mutant has no reported myogenic phenotype) and its ability to reprogram fibroblasts (Lee et al) to test by qPCR its expression at least for the Figure 2B. The legend of the Figure 6 must be modified, B/ heat maps...C/Venn diagram.

Figure 6C (Venn diagram presented) compares only FACS sorted cells from fetal W10, W18 and adult SC : cells that have not been expanded in culture. It would be interesting to show other Venn diagram in Supp with comparison with the 5 hPSC-SMPC cultures for which heat maps are presented. Have all data been deposited at the NCBI GEO omnibus?

The chapter from line 296 to 306 has many typo errors, and it is difficult to follow it. When the authors write: " ....may up regulate SIX1 to favor..." it seems that the contrary is described (down regulate SIX1). Also I would favor an alternative explanation/discussion introducing the notion of bi/multipotent transient cell fate of the reprogrammed hPSC (see for example DOI: 10.1242/dev.050674 , doi: 10.7554/eLife.70235) to explain their behavior, rather than the only cell-cell communication hypothesis that is presented (the two hypothesis are not exclusive). Down regulation of Isl1 (by sg/Cas9) for example in bipotent cardiac/skeletal progenitors would favor the acquisition of a skeletal myogenic fate, and increase their number. What the authors propose is that the downregulation of cardiac progenitors influence positively the number of skeletal myogenic progenitors, as if cardiac cells may secrete proteins that interfere negatively with skeletal myogenic fate acquisition. Although this last hypothesis is perhaps a good one, the other one must be discussed, as it is an important aspect during embryonic development.

© 2023. Published by The Company of Biologists under the terms of the Creative Commons Attribution License (https://creativecommons.org/licenses/by/4.0/).

In their last paragraph, the authors should identify in more details the expression of SIX1/2/4/5, EYA1/2/3/4 and PAX3/7 genes in the different samples analyzed, in the Venn diagrams, to link their hPSC studies with in vivo data of embryonic, fetal and adult myogenic stem cells.

I have a problem with many results showing two or three replicates with no statistical value, the authors must present and comment their results that show statistical significance, or explain the variation of their results, inherent to the culture conditions. If one aim of the study is to provide an improved reproducible protocol, this must be carefully addressed.

Line 352: (Figures 6A and S6): please correct Line 280: please correct the sentence by deleting "were required".

Line 327: " c-Met is now localized in the cytoplasm of ...". C-Met is a receptor localized at the cell membrane; difficult from the Figure 5D to confirm its membrane or cytoplasmic localization, and also difficult to assimilate c-Met as a "myogenic commitment factor".

Line 399, correct the sentence please Line 130-132, the reference Spokony et al does not show that absence of Sox9 and NCC cause skeletal muscle defects in Xenopus. Furthermore, initiation of Myf5 expression in branchial arches still takes place after ablation of CNCC in chick embryos (doi: 10.1242/dev.002501, doi: 10.1242/dev.02426). The relationship between NCC and myogenic cell fate acquisition must be better referenced.

Reviewer 2

Advance summary and potential significance to field

In this study the authors address a key challenge in using pluripotent cells for directed differentiation, namely the heterogeneity in response to defined culture conditions among different ES/iPS lines, a problem that appears to be more accentuated with human pluripotent cells. The authors set out to address this important issue by refining protocols and defining specific steps where different types of protocol modifications appear to generate more robust and predicable generation of myogenic cells. The authors highlight the importance of Wnt signaling (timing and cell density across tested lines) and provide compelling evidence for an emerging Pax3/Six1 population as a predictive readout for higher efficiency skeletal myogenesis as opposed to neuronal, chondrocyte or cardiomyocyte differentiation as alternative pathways. These experiments provide further refinement of existing protocols and better predictability for generating skeletal muscle progenitors from human pluripotent cells.

Comments for the author

Comments/suggestions, not in order of priority:
1) As a general comment, please indicate n-values for all experiments directly in the legends or on Figures.
2) Line 174 – "myotome to form the limb" … "… to form limb muscles."
3) Fig 1 legend has 2 "B" texts – please separate on figure as B and C to avoid confusion
4) The cMET staining in red appears to give a lot of background on the limb, and the region highlighted below contains more background staining than the upper part of this panel. Please provide control staining and highlight the more evident staining on the upper part of the section instead.
5) Although the authors indicate better co-staining of Pax3/Six1, these expression patterns on the single stained panels in Fig 1B look very different, and in the merged, the green Pax3 staining dominates. This co-staining of Pax3/Six1 is more convincing in Fig S1 – perhaps this should be shown instead. Please address this issue of co-expression. Also, does the Merge include DAPI staining? If so, please indicate in legend.
6) The authors focused on the coexpression of Pax3/Six1 however it is unclear to what extent they texted for Pax3/Pax7/Six1 as a complementary readout, or Paraxis as a dermomyotome marker (although Pax7 is also expressed in neuronal and neural crest cells).
7) Line 176 "pre-limb bud muscle progenitors"

© 2023. Published by The Company of Biologists under the terms of the Creative Commons Attribution License (https://creativecommons.org/licenses/by/4.0/).

8) Studies have emerged in the last year pointing to high mutation load in hiPSCs derived from fibroblasts, in particular, following extensive passaging. The table in Fig 2 shows hiPSCs with passages ranging from 16 to 49. Although passage number does not appear to correlate with Pax3 expression, the authors should nevertheless point out that some of the variability might be explained by the culture history of the iPSCs.
9) Line 193 – “showed higher levels of Pax3…”
10) Line 202 – the statement “MEOX1 cells may compete with PAX3 cells” is somewhat confusing as these two expression patterns seem to fully overlap in the limb Fig 1B.
11) Fig 3B - it is difficult to distinguish what is cellular material from these low-resolution images. Perhaps DAPI staining or another generic marker in parallel would clarify better.
12) Fig 4 and S5 – please indicate how were the reductions in expression obtained in each case. 40-60% repression represents a heterozygous state, so it is difficult to conclude from incomplete repression.
13) Line 299-301 – syntax problem
14) Line 321 – please explain rationale for using ERBB3/NGFR markers 15) Fig 5D – cMET receptor staining looks ambiguous as indicated for Fig 1. Please provide a control for this antibody.
16) Fig 5 indicates use of cells for transplantations. Did the authors perform this test, which has been done in many such studies from different labs in previous reports.
17) Fig 6 provides an RNAseq analysis and other than showing sample differences, it does not provide any satisfactory complementary information compared to the rest of the study. A better link with the experimental parts of the study would improve the quality of the manuscript.

First revision

Author response to reviewers' comments

Dear Dr. Bruneau,

We thank you and the referees for expressing interest in our work.

We have now addressed all reviewer comments, which includes a key new experiment that we find exciting and valuable. To summarize, we have identified an early myogenic population that arises 1 week into hPSC differentiation marked by SIX1 and PAX3 which can predict whether myogenic differentiation will be successful later on. To prove this, we used a dCas9-KRAB iPSC line to knockdown *SIX1* expression early during myogenic commitment and followed myogenic differentiation through 5 weeks. Early *SIX1* knockdown resulted in a massive decrease to myogenic differentiation and provides new mechanistic evidence for SIX1 regulation of myogenic development in hPSCs. The new experiments further address the reviewers’ key concern of finding a better link that ties the early week 1 experiments performed in this study with later week 5-6 experiments included in the RNA sequencing analysis.

The reviewers also requested additional replicate experiments, additional controls, revisions to the transcriptomic data, and revisions for references related to data interpretation which we have now completed. These revisions provide additional insight and alternative hypotheses to the conclusions that we originally interpreted, and further we have detailed the expression of SIX, EYA, and PAX genes across 3 stages of human development using our RNA sequencing datasets.

We hope that you will find the revised manuscript has addressed all reviewer concerns satisfactorily. We hope that this work will help multiple labs with their derivations of skeletal muscle from hPSCs. Please do not hesitate to contract us for any additional questions.

All the best Michael Hicks Mrhicks1@hs.uci.edu

Assistant Professor
Sue and Bill Gross Stem Cell Research Center
University of California, Irvine

© 2023. Published by The Company of Biologists under the terms of the Creative Commons Attribution License (https://creativecommons.org/licenses/by/4.0/).

Reviewer 1 Advance Summary and Potential Significance to Field:
The work of O.G. Jaime et al extends previous published works, by describing a detailed protocol for the culture of human pluripotent stem cells (hPSC) to induce their myogenic directed differentiation. The protocol is well detailed, and many steps are illustrated by the expression of Pax3, a gene suspected to drive the myogenic fate acquisition (activation of Myogenic regulatory factors) of these cultured cells, as already shown in vivo during mouse embryonic development, and by the expression of the myogenic stem cell marker Pax7. During this study the authors show at the RNA and protein-immunochemistry-imaging level that Pax3 and Six1 are co-expressed before Pax7 in cultured cells and represent the first emerging myogenic population. They study the reproducibility of their induction protocol in five distinct hPSC. They also show that application of CHIR to their culture can lead to distinct phenotype, depending on the manufacturer (SCT or Tocris). They further show that the culture medium although favoring myogenic fate acquisition, other cell types are present in their culture, among which Isl1+ cells, Sox9+ cells and Sox10+ cells. By reducing their expression by dCas9-KRAB the authors show by qPCR experiments that somehow, they favor the myogenic fate acquisition of their cultured hPSC. Results of bulk RNAseq experiments are presented at distinct times points of the kinetic and compared with embryonic, fetal and adult myogenic stem cells RNAseq.

Reviewer 1 Comments for the Author:
In light of the results presented, it would be interesting to discuss the manuscript (DOI: 10.1016/j.nmd.2022.04.006) showing that adult human SC express Pax7 and Six1 (or another manuscript showing coexpression of Six1/Pax7 in human myogenic stem cells), as well as the manuscript of Lee et al (DOI: 10.1038/s41419-018-1114-8) demonstrating that Pax3, Six1 and Eya1 can with Esrrb reprogram fibroblasts into a Pax7 myogenic progenitor cell fate.

We thank the reviewer for this comment and have now included both papers in the discussion. The Jensen et al., 2022 paper has now been referenced to show PAX7+SIX1+ identifies an adult satellite cell (SC) population and SIX1+ identifies the myonuclei and is supportive of our immunostaining data showing SIX1 expression by PAX7 cells and myotubes (Figures 4D and 6D). We have also incorporated the Lee at al., paper showing that Six1 over expression can reprogram fibroblasts into the myogenic lineage, and we have interpreted this to suggest that undifferentiated PAX3+ cells may require SIX1 for myogenic lineage commitment.

The authors must better explain the link between their first results describing the best culture ways to get robust PAX3+SIX1+ myogenic progenitors from hPSCs and the transcriptomic data generated.

We have put significant amount of work to link the first set of results describing how to generate better myogenic differentiations with the transcriptomic data at the final stages of myogenic differentiation described in Figures 6-7. This has been accomplished in two ways:

First, through the addition of a new Figure 4, we now show that in the absence of *SIX1*, human pluripotent stem cells (hPSCs) poorly differentiate into the myogenic lineage. We performed *SIX1* gRNA knockdown early during directed differentiation (days 4, 7, and 10) of hPSCs to skeletal muscle progenitor cells (SMPCs), and then evaluated the cultures for expression of SIX1+PAX7+ progenitors and Myosin+ myotubes later in differentiation (day 35). These results showed early knockdown of *SIX1* results in a significant loss of myogenic differentiation at later time points (Figure 4D).

Second, we have updated the transcriptomic data in Figure 7 to include several genes and co-factors that are involved with SIX1 or SIX1-related pathways. Interestingly, although SIX1 is expressed throughout human development and by adult satellite cells, we show that SIX1 expression levels change overtime, as well as many of its known cofactors such as EYA1-4 genes. We describe these changes in detail in the new results section and in the discussion to hone our message about SIX1 during myogenesis and its regulatory effects on hPSCs.

How have the cells been treated to get the hPSC-SMPC1-5 used for the transcriptomic experiments?
Transcriptomic data of hPSC SMPCs was collected after treatments described in the Differentiation

© 2023. Published by The Company of Biologists under the terms of the Creative Commons Attribution License (https://creativecommons.org/licenses/by/4.0/).

Procedure section of the methods and illustrated in Figure 6A. To make this clearer, the methods for generating hPSC SMPCs used for transcriptomic data are now updated in the paper.

In the Figure 6, replace in the transcription factors heat map SPRY1, 4 and Jak1 that are not transcription factors (and place them correctly in the Signaling heat map) by Eya2, Eya3 and Eya4 that may participate with Six1 to the myogenic progenitor fate of the induced hPSC.

We thank the reviewer for these points. SPRY1, 4 and Jak1 have now been removed and replaced from the transcription factor heatmap. The heatmaps in the original submission have been moved to Supplement Figure S6.

It would be also interesting due to the role of Eya1 in mouse embryonic development (be careful you wrote line 123 that mouse Eya2 mutant show down regulation of Pax3, but it is the double Eya1/Eya2 mutant that presents this phenotype in Grifone et al, 2007, the single Eya2 mutant has no reported myogenic phenotype) and its ability to reprogram fibroblasts (Lee et al) to test by qPCR its expression at least for the Figure 2B.

The Grifone et al., 2007 reference has now been carefully revised. We have also included the Lee et al., reference into the discussion. Although *EYA2* is not expressed early during directed differentiation, we have now tested for expression of all EYA and SIX genes in cultures that were screened for SIX1+PAX3+ cells. At early time points, *SIX1* is the highest expressed SIX gene relative to GAPDH. We also found *EYA1* was the highest expressed EYA gene. At the end of the directed differentiation, our transcriptomic data revealed that all EYA genes were expressed by hPSC SMPCs, and we also found that human fetal week 18 and adult SCs only express *EYA3/4*. These results have been updated in Figure 7C and used to link the first results describing the best culture ways to get robust PAX3+SIX1+ myogenic progenitors from hPSCs with the transcriptomic data generated.

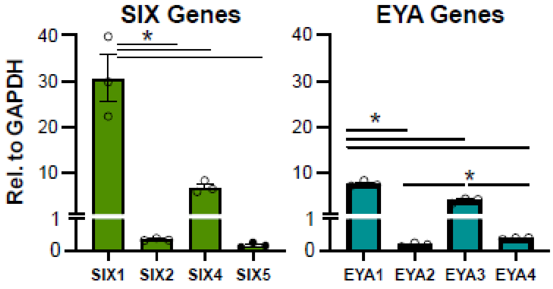

Figure shows SIX and EYA gene expression relative to GAPDH at day 9 of directed differentiation.

The legend of Figure 6 must be modified, B/ heat maps...C/Venn diagram.

This legend now in Figure 7 and S6 have now been corrected.

Figure 6C (Venn diagram presented) compares only FACS sorted cells from fetal W10, W18 and adult SC: cells that have not been expanded in culture. It would be interesting to show another Venn diagram in Supp with comparison with the 5 hPSC-SMPC cultures for which heat maps are presented.

The Venn Diagram (originally Figure 6C) has now been moved to Supplement Figure S6 to make space for data that better link the first results describing the best cultures with the transcriptomic data generated. The Venn Diagram has been updated to show comparisons of all genes upregulated by fetal SMPCs and adult SCs compared to hPSC SMPCs. We have also included a second Venn Diagram showing in the Supplement Figure S6 showing all genes upregulated by adult SCs compared to fetal and hPSC SMPCs.

Has all data been deposited at the NCBI GEO omnibus?
Our data has now been submitted to the NCBI GEO omnibus (Ref #:35277545). We have included all original FASTQ files, a metadata spreadsheet, the table of gene counts and differentially expressed genes, and a summary describing how to best use these data which is also updated in the methods

© 2023. Published by The Company of Biologists under the terms of the Creative Commons Attribution License (https://creativecommons.org/licenses/by/4.0/).

section. The GEO data is set to be released with this paper.

The chapter from line 296 to 306 has many typo errors, and it is difficult to follow it. When the authors write: “ ....may up regulate SIX1 to favor...” it seems that the contrary is described (downregulate SIX1). Also I would favor an alternative explanation/discussion introducing the notion of bi/multipotent transient cell fate of the reprogrammed hPSC (see for example DOI: 10.1242/dev.050674 , doi: 10.7554/eLife.70235) to explain their behavior, rather than the only cell-cell communication hypothesis that is presented (the two hypothesis are not exclusive). Down regulation of Isl1 (by sg/Cas9) for example in bipotent cardiac/skeletal progenitors would favor the acquisition of a skeletal myogenic fate, and increase their number. What the authors propose is that the downregulation of cardiac progenitors positively influences the number of skeletal myogenic progenitors, as if cardiac cells may secrete proteins that interfere negatively with skeletal myogenic fate acquisition. Although this last hypothesis is perhaps a good one, the other one must be discussed, as it is an important aspect during embryonic development.

We thank the reviewers for the interesting point on multipotent progenitors. We agree that upon mesoderm induction, we may be deriving progenitors with multipotent properties in addition to differentiating lineage specific cell types. We now included this point in the second to last paragraph of the discussion. We have focused on PAX3+ expressing cells and hypothesize that these cells may have multipotent properties. In Figure 1C, we show that PAX3+ cells can be co- expressed with SIX1+ or with MEOX1+. In Figure 2C, we show that PAX3 by itself does not identify myogenic progenitors as it can also be co-expressed with SOX10+, and in supplemental figure 2C, we demonstrate that poor hPSC maintenance drives a PAX3+PAX6+ progenitor. These data suggest that like embryonic development, direct differentiation to muscle from hPSCs may also generate progenitors with multipotent cell fates. Although myogenic and cardiac cells may arise from a common progenitor or compete with each other, we have removed cardiac lineages from the discussion since we did not provide enough evidence of late stages of cardiac differentiation and based on our data, we focused on the hypothesis that PAX3 cells may have multipotent potential.

In their last paragraph, the authors should identify in more details the expression of SIX1/2/4/5, EYA1/2/3/4 and PAX3/7 genes in the different samples analyzed, in the Venn diagrams, to link their hPSC studies with in vivo data of embryonic, fetal and adult myogenic stem cells.

Per the reviewer’s suggestion, we have now edited the last paragraph to include details the expression of SIX1/2/4/5, EYA1/2/3/4 and PAX3/7 genes in the different samples analyzed. We have also now highlighted these genes in a new Figure 7B-C which shows transcriptomic count data and significance using the false discovery rate (FDR) <0.05 from our RNA-seq analysis.

I have a problem with many results showing two or three replicates with no statistical value, the authors must present and comment on their results that show statistical significance, or explain the variation of their results, inherent to the culture conditions. If one aim of the study is to provide an improved reproducible protocol, this must be carefully addressed.

We thank the reviewer for this critique. We have now performed statistical analysis (either t- tests or one-way ANOVA with multiple comparisons) on all experiments containing an n=3 or more biological replicates. The results have been specified to show significant differences via asterixis in the graphs and P values are shown on the figure legends.

For Figure 2B, we performed one-way ANOVA without multiple comparisons which shows significant differences between groups in PAX3, SIX1, MEOX1, and EYA2, as denoted by *. Multiple comparison analysis was omitted from this figure because our point was to show differences between hPSC lines, and not which line had the highest expression, as the purpose was to show line-to-line variability.

Line 352: (Figures 6A and S6): please correct
This line now pertains to the now Figure 7 and supplemental figure 6 and corrected to (Figure 7A and S6).

Line 280: please correct the sentence by deleting “were required”
We have now corrected the sentence by deleting the “were required” phrase.

© 2023. Published by The Company of Biologists under the terms of the Creative Commons Attribution License (https://creativecommons.org/licenses/by/4.0/).

Line 327: " c-Met is now localized in the cytoplasm of ...". C-Met is a receptor localized at the cell membrane; difficult from the Figure 5D to confirm its membrane or cytoplasmic localization, and also difficult to assimilate c-Met as a "myogenic commitment factor".

We agree c-Met is a receptor expressed on the cell membrane, and this has been corrected in the text. We have also removed from the text that cMET is a myogenic commitment factor. We do point out, that cMET has a striking similar expression pattern as MF20, potentially demonstrating that cMET is expressed by myotubes later during their differentiation. Below is an image taken from Figure 6D of myotubes differentiated from hPSC-derived SMPCs and stained for Hoechst (blue), MF20 (red) and cMET (magenta).

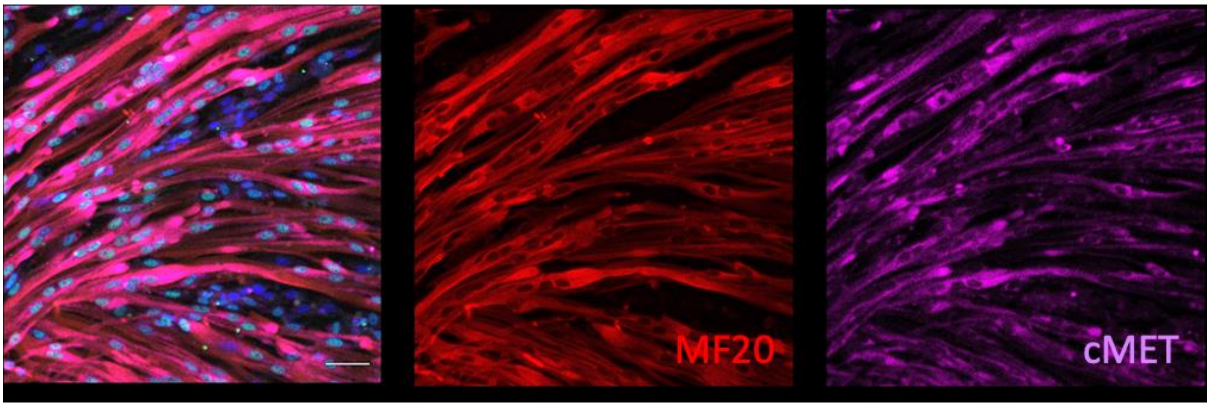

Line 399, correct the sentence please
The sentence has been corrected.

Line 130-132, the reference Spokony et al does not show that absence of Sox9 and NCC cause skeletal muscle defects in Xenopus. Furthermore, initiation of Myf5 expression in branchial arches still takes place after ablation of CNCC in chick embryos (doi: 10.1242/dev.002501, doi: 10.1242/dev.02426). The relationship between NCC and myogenic cell fate acquisition must be better referenced.

The reference has now been updated to highlight that pre-chondrocytes in limb development have roles in skeletal muscle formation. The new reference by Akiyama et al., 2002 shows that the Sox9 mutant limb buds do not form correctly, and organization of muscle bundles were very abnormal.

Reviewer 2 Advance Summary and Potential Significance to Field:
In this study the authors address a key challenge in using pluripotent cells for directed differentiation, namely the heterogeneity in response to defined culture conditions among different hESC and hiPSC lines, a problem that appears to be more accentuated with human pluripotent cells. The authors set out to address this important issue by refining protocols and defining specific steps where different types of protocol modifications appear to generate more robust and predicable generation of myogenic cells. The authors highlight the importance of Wnt signaling (timing and cell density across tested lines) and provide compelling evidence for an emerging Pax3/Six1 population as a predictive readout for higher efficiency skeletal myogenesis as opposed to neuronal, chondrocyte or cardiomyocyte differentiation as alternative pathways. These experiments provide further refinement of existing protocols and better predictability for generating skeletal muscle progenitors from human pluripotent cells.

Reviewer 2 Comments for the Author:
Comments/suggestions, not in order of priority:

1)As a general comment, please indicate n-values for all experiments directly in the legends or on Figures.
We thank you for this reminder. We have now included n-values for all experiments directly in the legends and have performed statistical analysis for all experiments containing an n=3 or more.

© 2023. Published by The Company of Biologists under the terms of the Creative Commons Attribution License (https://creativecommons.org/licenses/by/4.0/).

2)Line 174 – "myotome to form the limb" … "… to form limb muscles."
Line 174 has now been corrected to read "myogenic progenitors have delaminated from the myotome to form limb muscles.

3)Fig 1 legend has 2 "B" texts – please separate on figure as B and C to avoid confusion
Figure 1 legend has now been corrected to specify the human limb as Figure 1B and hPSC- derived cells as figure 1C.

4)The cMET staining in red appears to give a lot of background on the limb, and the region highlighted below contains more background staining than the upper part of this panel. Please provide control staining and highlight the more evident staining on the upper part of the section instead.
We thank the reviewers for this comment, the cMET stain may have appeared non-specific. The embryonic week 7 tissue was a bit damaged, we were provided a second embryonic week 6.5 human tissue and performed re-staining of cMET. Our data find that the cMET receptor is expressed on the nuclear membrane and is expressed by PAX3 cells and other cells of the limb mesenchyme (Figure 1B).

Per the reviewer's request, we have also provided control staining on embryonic week 7 tissues. Below in A) we provide fluorescent images of the limb stained with Hoechst (blue), PAX3 (green), and cMET (red), and in B) we provide an isotype control for both PAX3 and cMET antibodies and include a second control for the isotype specific HRP antibodies with the primary antibody omitted (scale bar = 100µm). In the control images, we used arrows to highlight the non-specific and background fluorescence of red blood cells obtained from both controls, which are distinct from the true cMET and PAX3 staining. We did not include these control images in the paper but would be happy to include in the supplement if suggested by the reviewer.

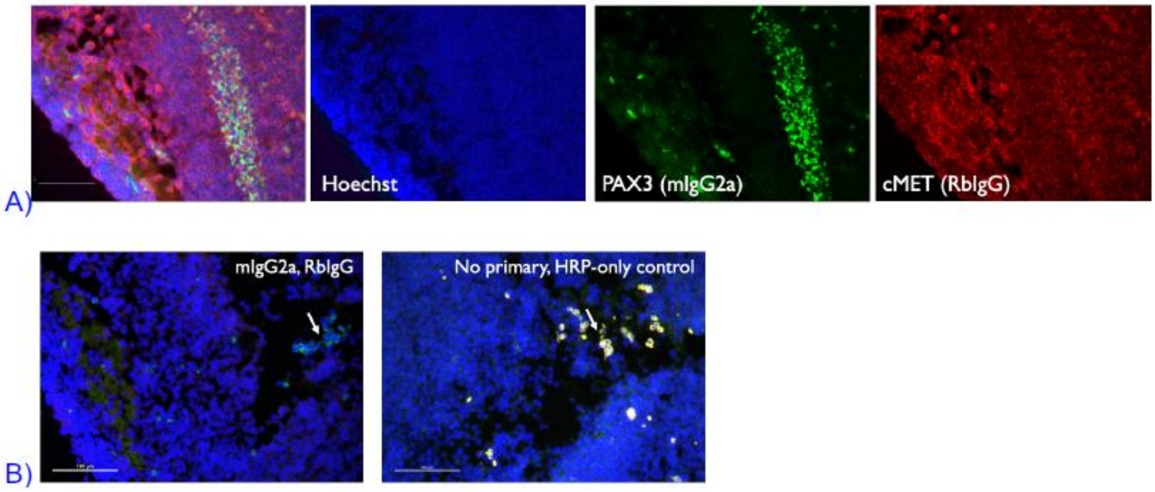

5)Although the authors indicate better co-staining of Pax3/Six1, these expression patterns on the single stained panels in Fig 1B look very different, and in the MERGED, the green Pax3 staining dominates. This co-staining of Pax3/Six1 is more convincing in Fig S1 – perhaps this should be shown instead. Please address this issue of co-expression. Also, does the Merge include DAPI staining? If so, please indicate in legend.
We thank you for pointing this out. For clarity, in Figure 1C, we provide new images showing three PAX3 clusters that are also positive for SIX1 to highlight the double positive SIX1+PAX3+ progenitor that arises during myogenic differentiation of hPSCs. We have now indicated the Hoechst stain in the figure legend. We agree that Figure S1 is also clear shows double positive SIX1+PAX3+ cells; however, this data was included to show that this myogenic population arises from multiple directed differentiation approaches (Chal et al., 2016).

6)The authors focused on the coexpression of Pax3/Six1 however it is unclear to what extent they texted for Pax3/Pax7/Six1 as a complementary readout, or Paraxis as a dermomyotome marker

© 2023. Published by The Company of Biologists under the terms of the Creative Commons Attribution License (https://creativecommons.org/licenses/by/4.0/).

(although Pax7 is also expressed in neuronal and neural crest cells).

We thank the reviewer for pointing this out. In our early time point differentiation studies, we find that *PAX7* is not expressed by SMPCs (Day 9), as we were unable to detect it by immunostaining. The earliest that we performed rt-PCR and immunostaining for PAX7 was at day 35, and at that time point PAX7 is co-expressed with SIX1 (new Figure 4D).

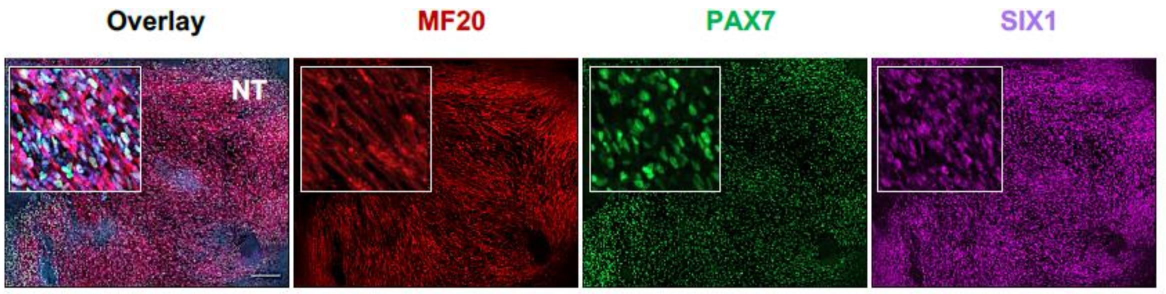

We have performed multiple tests for expression of somite and dermomyotome markers by Rt-qPCR which have been described in the literature such as *SNAI1, LBX1, BRAF, DACH1, EN1, TCF15, TBX6, and MSGN1*. We do find some expression of SNAI1 and EN1 but low or no expression of the other genes at day 9 which may suggest that cells have differentiated beyond the paraxial mesoderm or somite stage. However, we have yet to fully define dermomyotome markers from hPSCs. As the reviewer has asked about Paraxis (*TCF15*) we have included this data below which shows that at the time we evaluated, *TCF15* was not expressed at levels greater than the day 0, human pluripotent stem cell colonies. We also looked at TCF15 in our RNA-seq datasets and this gene was not expressed in human fetal week 9-20 data.

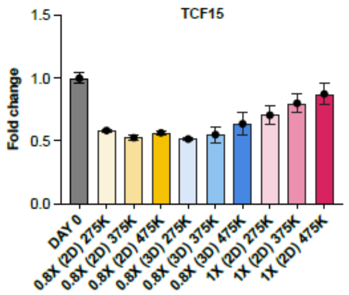

Rt-qPCR expression of TCF15 at 9 days of differentiation compared to day 0 hPSCs. The seeding densities (275K-475K) and CHIR concentrations (0.8X-1.0X) for 2 days (2D) or 3 days (3D) are shown. This gene was not expressed.

8)Studies have emerged in the last year pointing to high mutation load in hiPSCs derived from fibroblasts, in particular, following extensive passaging. The table in Fig 2 shows hiPSCs with passages ranging from 16 to 49. Although passage number does not appear to correlate with Pax3 expression, the authors should nevertheless point out that some of the variability might be explained by the culture history of the iPSCs.
This is a valid point, thank you for bringing it up. We agree that mutations which occur during cell division can lead to selective advantages in cell growth, such as through BCL2 mutations. During this study, we performed Karyotyping on different cell lines or passage numbers using Cell Line Genetics. This did result in our lab throwing a passage 56 H9 clone with a BCL2L1 mutation, but this line was not included in the manuscript. The lines that we have included did not have abnormalities.

We were able to generate SIX1+PAX3+ positive cells from multiple hESC and hiPSC lines regardless of their passage number. We do find that abnormalities can occur especially from user variability such as from over confluence. We have shown in Figure S2C that when poor maintenance of iPSCs results in skewed neuronal progenitor cell fate differentiation rather than the myogenic lineage.

9)Line 193 – "showed higher levels of Pax3…"
This sentence has now been corrected.

10)Line 202 – the statement "MEOX1 cells may compete with PAX3 cells" is somewhat confusing as

© 2023. Published by The Company of Biologists under the terms of the Creative Commons Attribution License (https://creativecommons.org/licenses/by/4.0/).

these two expression patterns seem to fully overlap in the limb Fig 1B
This statement about cell competition was removed. In the discussion we propose that PAX3+MEOX1+ cells have multipotent potential per reviewer 1 suggestion.

11)Fig 3B – it is difficult to distinguish what cellular material is from these low-resolution images. Perhaps DAPI staining or another generic marker in parallel would clarify better.
We point out in Figure 3B the distinct morphology of the cultures generated by optimizing seeding density and concentrations of CHIR. We receive numerous emails from groups seeking to reproduce our protocol and we wanted to emphasize the bright field images which allows users to reproduce our work following a non-destructive approach via live imaging (this is also why we have included the non-destructive lactate secretion assays in Figure S3). We wanted to highlight the importance of the three-dimensional structures that arise as an early indication of the rising SIX1+PAX3+ cell as shown by arrowheads and adding DAPI or another genetic marker requires fixation and therefore would not allow users to continue differentiations. Arrowheads of 3D structures in optimized differentiations are also shown in the new Figure 4B which corresponds to highly efficient differentiations shown in new Figure 4C.

12)Fig 4 and S5 – please indicate how the reductions in expression were obtained in each case. 40-60% repression represents a heterozygous state, so it is difficult to conclude from incomplete repression.
We thank the reviewer for this comment and have added this point as a limitation in the discussion. Because directed differentiations have been optimized in a way that does not allow for their dissociation at early time points, we were unable to apply more efficient transfection methods such as nucleofection; rather we were limited to perform Lipofection. We tested multiple Lipofection approaches including Lipo-Stem and Lipofectin made by Dr. Phil Felgner (UCI scientist who developed Lipofection, DNA transfection technology). Based on our preliminary data using Lipofectin and a P-max plasmid, we expect that the incomplete repression in directed differentiation cultures is due to the transfection efficiency. We found that not all cells transfected with the P-Max plasmid as measured by GFP. We found that GFP was highest expressed in earlier transfected cultures i.e., days 3-5 rather than later cultures i.e., days 7-10. This is complicated because our rtPCR data show that some of the genes do not turn on until the 7-10 day time point. The gRNAs (100 base pairs) are smaller than the pMax plasmid (3000 base pairs), so we anticipated that these would transfect better than pMax. However, this is a limitation of the study that we have mentioned in the paper. To overcome this limitation, we performed a series of 2 Lipofections at days 4 and 7 for cultures that ended early at day 9, and we performed 3 Lipofections at day 4, 7, and 10 for cultures that continued until day 36. Secondly, we added multiple gRNAs targeting both the promoter and transcriptional start sites.

13)Line 299-301 – syntax problem
We have now revised the syntax throughout the manuscript.

14)Line 321 – please explain rationale for using ERBB3/NGFR markers
These markers were previously identified by our lab and are now referenced in the introduction and results section (Hicks et al., 2018). We have now included a new Figure that shows RT- qPCR of muscle cells enriched for the ERBB3 and NGFR markers, compared to cells negative for these markers. We show a >100-fold increase in PAX7 and >50 fold increase in SIX1 when SMPCs are enriched for ERBB3 and NGFR compared to double negative populations.

15)Fig 5D – cMET receptor staining looks ambiguous as indicated for Fig 1. Please provide a control for this antibody.
We thank the reviewer for pointing this out. Our cMET antibody is a RbIgG and below, we provide same day staining images of A) fetal SMPC-derived myotubes stained with cMET (magenta) and also provide staining for MF20 to emphasize its' expression similarity. Similar to MF20, cMET appears to be expressed by myotubes. In B) are isotype controls for MF20 (mIgG2b) and cMET (RbIgG). The isotype control for cMET, shows minimal non-specific expression and confirms true cMET expression by myotubes. Isotype control images were omitted from the paper, but we could include these in the supplement if required.

© 2023. Published by The Company of Biologists under the terms of the Creative Commons Attribution License (https://creativecommons.org/licenses/by/4.0/).

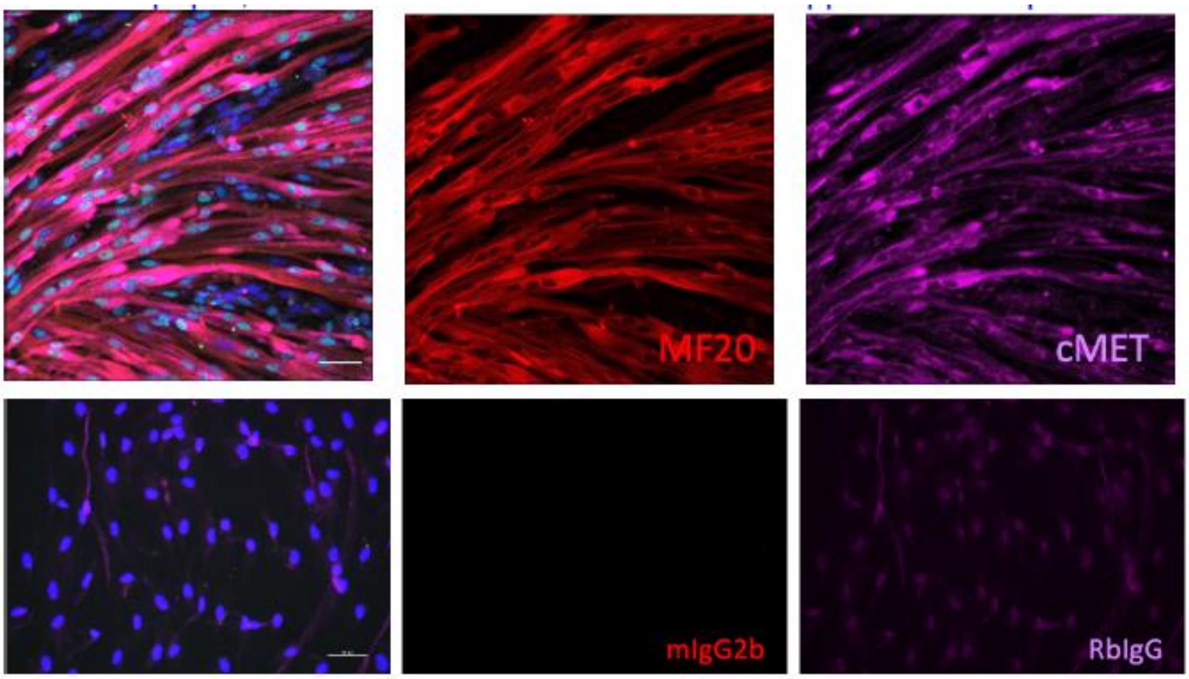

16)Fig 5 indicates use of cells for transplantations. Did the authors perform this test, which has been done in many such studies from different labs in previous reports.

The focus of this study was to improve myogenic differentiation by examining line to line variability and identifying early markers of hPSC myogenic commitment. We have revised the diagram on the now Figure 6 to remove any misinterpretations that we performed transplantations in this study. Our laboratory has performed several cell transplantations from iPSCs using this protocol, but these were outside the scope of this current study.

17)Fig 6 provides an RNAseq analysis and other than showing sample differences, it does not provide any satisfactory complementary information compared to the rest of the study. A better link with the experimental parts of the study would improve the quality of the manuscript.

Both Reviewers 1 and 2 came to the same conclusion related to the RNA seq analysis and thus we have significantly revised this dataset.

We have put significant amount of work to link the first set of results describing how to generate better myogenic differentiations with the transcriptomic data at the final stages of myogenic differentiation described in Figures 6-7. This has been accomplished in two ways:

First, through the addition of a new Figure 4, we now show that in the absence of *SIX1*, human pluripotent stem cells (hPSCs) poorly differentiate into the myogenic lineage. We performed *SIX1* gRNA knockdown early during directed differentiation (days 4, 7, and 10) of hPSCs to skeletal muscle progenitor cells (SMPCs), and then evaluated the cultures for expression of SIX1+PAX7+ progenitors and Myosin+ myotubes later in differentiation (day 35). These results showed early knockdown of *SIX1* results in a significant loss of myogenic differentiation at later time points (Figure 4D).

Second, we have updated the transcriptomic data in Figure 7 to include several genes and co-factors that are involved with SIX1 or SIX1-related pathways. Interestingly, although SIX1 is expressed throughout human development and by adult satellite cells, we show that SIX1 expression levels change overtime, as well as many of its known cofactors such as EYA1-4 genes. We describe these changes in detail in the new results section and in the discussion to hone our message about SIX1 during myogenesis and its regulatory effects on hPSCs.

© 2023. Published by The Company of Biologists under the terms of the Creative Commons Attribution License (https://creativecommons.org/licenses/by/4.0/).

Second decision letter

MS ID#: DEVELOP/2022/201509

MS TITLE: SIX1+PAX3+ Identify a Progenitor for Myogenic Lineage Commitment from hPSCs

AUTHORS: Olga G Jaime, Jessica Arias, Shreya Pavani, April D Pyle, and Michael R Hicks

I have now received all the referees reports on the above manuscript, and have reached a decision. The referees' comments are appended below, or you can access them online: please go to BenchPress and click on the 'Manuscripts with Decisions' queue in the Author Area.

The overall evaluation is positive and we would like to publish a revised manuscript in Development, provided that the referees' comments can be satisfactorily addressed. Please attend to all of the reviewers' comments in your revised manuscript and detail them in your point-by-point response. If you do not agree with any of their criticisms or suggestions explain clearly why this is so. If it would be helpful, you are welcome to contact us to discuss your revision in greater detail. Please send us a point-by-point response indicating your plans for addressing the referee's comments, and we will look over this and provide further guidance.

Reviewer 1

Advance summary and potential significance to field

The work of O.G. Jaime et al extends previous published works, by describing a detailed protocol for the culture of human pluripotent stem cells (hPSC) to induce their myogenic directed differentiation. The protocol is well detailed, and many steps are illustrated by the expression of Pax3, a gene suspected to drive the myogenic fate acquisition (activation of Myogenic regulatory factors) of these cultured cells, as already shown in vivo during mouse embryonic development, and by the expression of the myogenic stem cell marker Pax7. During this study the authors show at the RNA and protein-immunochemistry-imaging level that Pax3 and Six1 are coexpressed before Pax7 in cultured cells and represent the first emerging myogenic population. They study the reproducibility of their induction protocol in distinct hPSC. They also show that application of CHIR to their culture can lead to distinct phenotype, depending of the manufacturer (SCT or Tocris). They further show that the culture medium although favoring myogenic fate acquisition, other cell types are present in their culture, among which Isl1+ cells, Sox9+ cells and Sox10+ cells. By reducing the expression of Six1 by dCas9-KRAB the authors show by immunofluorescence the reduction of Pax7 and Mf20 expression, indicating myogenic fate acquisition decrease, and highlighting the requirement of Six1+ Pax3 to drive myogenesis in hPSC.

Comments for the author

The manuscript of Jaime et al has been modified, and greatly improved. The authors have responded to the comments made, and the manuscript has achieved credibility on the addressed topic. I still have some minor points.

In the attached procedure for culturing hPSCs:
- it is mentioned on page 4 to add 2 microliters of human Fc block in point 6. Can the authors clarify?
- and in point 8 the addition of 2 microliters per million cells of ERBB3, NGFR and HNK1. Obviously there is an error, because the authors sort their ERBB3+, NGFR+, HNK1- cells. Please correct or explain.
- and in Page 2 , should be optimized instead of "optimize.d"

In the main text:

© 2023. Published by The Company of Biologists under the terms of the Creative Commons Attribution License (https://creativecommons.org/licenses/by/4.0/).

Page 13, be careful to interpret that isl1+ positive cells are cardiac cells, without other markers. There are also craniofacial mesodermal cells that are isl1+ that will give skeletal muscle progenitors (DOI: 10.1016/j.ceb.2021.06.005).
In Sup6A, could the heat map have the same organization than the heat map in Fig7B for clarity and easy comparison: same order of cell types in the y-axis and genes in the x-axis?

Reviewer 2

Advance summary and potential significance to field

Comments for the author

I would go ahead with it and am generally satisfied, but I am not convinced by the anti-Met staining in vivo and in vitro, as this is a tricky antibody and it needs to be tested on the null to be sure. I am reluctant to add more work to what was already done, but am concerned that the staining is misleading. If the authors can produce an RNAscope or similar staining that would be more convincing.

Second revision

Author response to reviewers' comments

Reviewer 1 Advance Summary and Potential Significance to Field:
The work of O.G. Jaime et al extends previous published works, by describing a detailed protocol for the culture of human pluripotent stem cells (hPSC) to induce their myogenic directed differentiation. The protocol is well detailed, and many steps are illustrated by the expression of Pax3, a gene suspected to drive the myogenic fate acquisition (activation of Myogenic regulatory factors) of these cultured cells, as already shown in vivo during mouse embryonic development, and by the expression of the myogenic stem cell marker Pax7. During this study the authors show at the RNA and protein-immunochemistry-imaging level that Pax3 and Six1 are co-expressed before Pax7 in cultured cells and represent the first emerging myogenic population. They study the reproducibility of their induction protocol in distinct hPSC. They also show that application of CHIR to their culture can lead to distinct phenotype, depending of the manufacturer (SCT or Tocris). They further show that the culture medium although favoring myogenic fate acquisition, other cell types are present in their culture, among which Isl1+ cells, Sox9+ cells and Sox10+ cells. By reducing the expression of Six1 by dCas9-KRAB the authors show by immunofluorescence the reduction of Pax7 and Mf20 expression, indicating myogenic fate acquisition decrease, and highlighting the requirement of Six1+ Pax3 to drive myogenesis in hPSC.

Reviewer 1 Comments for the Author:
The manuscript of Jaime et al has been modified, and greatly improved. The authors have responded to the comments made, and the manuscript has achieved credibility on the addressed topic.

I still have some minor points.
In the attached procedure for culturing hPSCs:
- it is mentioned on page 4 to add 2 microliters of human Fc block in point 6. Can the authors clarify?
Fc Block is a commonly used reagent that is included in flow cytometry experiments to prevent non-specific binding. This step is performed prior to staining of our antibodies to saturate Fc receptors and prevent background or false positive signal. Blocking antibody was used at an optimized concentration of 2ul/1x10^6 cells to block non-specific binding to Fc receptors during flow cytometry staining.

- and in point 8 the addition of 2 microliters per million cells of ERBB3, NGFR and HNK1.

© 2023. Published by The Company of Biologists under the terms of the Creative Commons Attribution License (https://creativecommons.org/licenses/by/4.0/).

Obviously, there is an error because the authors sort their ERBB3+, NGFR+, HNK1- cells. Please correct or explain.
ERBB3, NGFR, and HNK1 are receptors expressed by cells in heterogenous hPSC-derived cultures. We used flow cytometry antibodies at a concentration of 2 microliters per million cells to label these receptors. Cells that are positive for ERBB3 and NGFR, and negative for HNK1 best isolate PAX7+ myogenic progenitors.

- and in Page 2, should be optimized instead of “optimize.d”
Thank you and good catch, the sentence has been corrected.

In the main text:
Page 13, be careful to interpret that isl1+ positive cells are cardiac cells, without other markers. There are also craniofacial mesodermal cells that are isl1+ that will give skeletal muscle progenitors (DOI: 10.1016/j.ceb.2021.06.005).
We thank the reviewer for this point that the ISL1+ cells may identify other mesodermal progenitors in addition to early cardiomyocytes. We have removed the word cardiac from ISL1+ expressing cells from this section.

In Sup6A, could the heat map have the same organization than the heat map in Fig7B for clarity and easy comparison: same order of cell types in the y-axis and genes in the x-axis?
We thank the reviewer for this point. We have re-formatted the graphs in Sup6A to stay consistent with the formatting of the RNA-seq graphs in Fig7B.

Reviewer 2 Advance Summary and Potential Significance to Field:

Reviewer 2 Comments for the Author:
I would go ahead with it and am generally satisfied, but I am not convinced by the anti-Met staining in vivo and in vitro, as this is a tricky antibody, and it needs to be tested on the null to be sure. I am reluctant to add more work to what was already done but am concerned that the staining is misleading. If the authors can produce an RNAscope or similar staining that would be more convincing.

We did test our c-MET on a null group. The null group was already included in the manuscript Figure 1C which showed no staining of day 9 hPSC-derived cultured. We used the same cMET antibody for staining cells and tissues, and we found that its’ expression was selective to only some cells such as the cultures in Figure 6D.

To further highlight this point, we have provided additional cMET stainings below. (**A**) We did not find non-specific fluorescent staining in the HRP and Isotype controls. (**B**) cMET was absent in the null day 9 hPSC-derived pre- myogenic cultures. (**C**) c-MET was expressed by fetal SMPCs derived myotubes in culture and by (**D**) cells of the fetal limb. We hope that these data can serve as a reassurance of our cMET antibody tests.

© 2023. Published by The Company of Biologists under the terms of the Creative Commons Attribution License (https://creativecommons.org/licenses/by/4.0/).

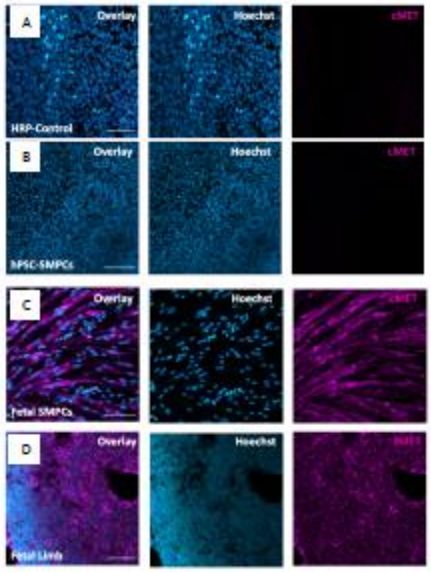

Third decision letter

MS ID#: DEVELOP/2022/201509

MS TITLE: SIX1+PAX3+ identify a progenitor for myogenic lineage commitment from hPSCs

AUTHORS: Olga G. Jaime, Jessica Arias, Shreya Pavani, April D. Pyle, and Michael R. Hicks
ARTICLE TYPE: Techniques and Resources Article

I am happy to tell you that your manuscript has been accepted for publication in Development, pending our standard ethics checks.

© 2023. Published by The Company of Biologists under the terms of the Creative Commons Attribution License (https://creativecommons.org/licenses/by/4.0/).
